# Defining alteration in bone marrow mesenchymal stem cells (MSC) from acute myeloid leukemia and exploring cultured MSC-conditioned media as a novel anti-leukemia therapy agent

**DOI:** 10.1007/s00262-025-04262-2

**Published:** 2026-02-23

**Authors:** Manasi Nagare, Monalisa Sahoo, Manju Sengar, Sachin Punatar, Navin Khattry, Anant Gokarn, Bhausaheb Bagal, Hasmukh Jain, Sumeet Mirgh, Sridhar Epari, Tanuja Shet, Trupti Pradhan, Shweta Shirsat, Madan Barkume, Caroline Mathen, Poonam Gera, Rohit Kumar Verma, Elveera Saldanha, Pratik Chandrani, Jitendra Gawde, Shubhada Chiplunkar, Jyoti Kode

**Affiliations:** 1 Kode Lab, Tumor Immunology and Immunotherapy Group, Advanced Centre for Treatment, Research and Education in Cancer, Tata Memorial Centre, Kharghar, Navi Mumbai, 410210 India; 2 Anticancer Drug Screening Facility (ACDSF), Advanced Centre for Treatment, Research and Education in Cancer, Tata Memorial Centre, Kharghar, Navi Mumbai, 410210 India; 3 Department of Medical Oncology, Advanced Centre for Treatment, Research and Education in Cancer, Tata Memorial Centre, Kharghar, Navi Mumbai, 410210 India; 4 Department of Pathology, Advanced Centre for Treatment, Research and Education in Cancer, Tata Memorial Centre, Kharghar, Navi Mumbai, 410210 India; 5 Chiplunkar Lab, Tumor Immunology and Immunotherapy Group, Advanced Centre for Treatment, Research and Education in Cancer, Tata Memorial Centre, Kharghar, Navi Mumbai, 410210 India; 6 Computational Biology, Bioinformatics and Crosstalk Lab, Advanced Centre for Treatment, Research and Education in Cancer, Tata Memorial Centre, Kharghar, Navi Mumbai, 410210 India; 7 Biorepository Department, Advanced Centre for Treatment, Research and Education in Cancer, Tata Memorial Centre, Kharghar, Navi Mumbai, 410210 India; 8 Biostatistics, Cancer Research Institute, Advanced Centre for Treatment, Research and Education in Cancer, Tata Memorial Centre, Kharghar, Navi Mumbai, 410210 India; 9 Department of Pathology, Tata Memorial Hospital, Tata Memorial Centre, Mumbai, 400012 India; 10 Homi Bhabha National Institute (HBNI), Training School Complex, Anushakti Nagar, Mumbai, 400094 India; 11 OCT Therapies and Research Private Limited, Bhandup Village, Bhandup (W), Mumbai, 400078 India

**Keywords:** Acute myeloid leukemia, Bone marrow microenvironment, Mesenchymal stem cells, Patient-derived MSC-conditioned media, In vivo anticancer efficacy, Inflammasome pathway regulation

## Abstract

**Background:**

Bone marrow-resident mesenchymal stem cells (BM-MSC) often play a role in acute myeloid leukemia (AML) progression and drug resistance by exerting immunomodulatory effects on cellular as well as soluble milieu. The current study aimed to understand the dynamic interplay between AML-BM-MSC and their soluble factors in determining the fate of AML blasts within the microenvironment.

**Methods:**

AML-BM-MSC were cultured and characterized for expression of phenotyping markers, multilineage differentiation potential, and gene expression analysis by microarray. To understand cross talk, AML-BM-MSC were co-cultured with the AML cell line OCI-AML2 and assessed for changes in cell cycle phases, mitochondrial activity, and cytarabine-induced cell death. Differential regulation of AML blast fate was evaluated by conducting co-culture experiments with cell-free-conditioned media (PD-MSC-CM) and in the presence of cell–cell contact of AML-BM-MSC. Further, PD-MSC-CM was assessed in vivo for tumor reduction potential using a leukemia xenograft model.

**Results:**

Besides standard features, AML-BM-MSC exhibited increased vesicles, MSC bodies (exosomes), and mitochondria. Altered AML-BM-MSC demonstrated upregulation of inflammasome pathway markers in microarray, which was further validated by ELISA and quantitative real-time polymerase chain reaction. Co-culture experiments on AML-BM-MSC revealed protective effects on AML blasts in the presence of cytarabine. In contrast, PD-MSC-CM significantly inhibited AML cell growth alone and synergistically with cytarabine. Further, PD-MSC-CM significantly reduced tumor growth in the leukemia mouse model, and this effect was mediated by regulation of the NLRP3 inflammasome pathway.

**Conclusion:**

Summarizing, the leukemic blasts and AML-MSC in the BM microenvironment interact differentially in cell–cell contact compared to only soluble factors. Further, our study has provided innovative leads that PD-MSC-CM effectively abrogates leukemia tumor growth, enhances chemosensitivity and can be developed further as an immunomodulatory novel "off-the-shelf" therapeutic agent for leukemia.

**Supplementary Information:**

The online version contains supplementary material available at 10.1007/s00262-025-04262-2.

## Introduction

Acute myeloid leukemia (AML) is an aggressive neoplasia of myeloid lineage, predominantly affecting adults. Developing leukemic blasts rapidly colonize the bone marrow, thus depleting the number of differentiated mature blood cells. The bone marrow (BM) niche comprises a complex array of cells (hematopoietic and stromal) and their secretory constituents. There exists a constant state of bidirectional interactions among the cellular and soluble components to maintain a healthy, quiescent pool of hematopoietic stem cells (HSC), regulate their differentiation and proliferation in response to stimuli and maintain homeostasis within the BM microenvironment [1–5]. Mesenchymal stem cells (MSC) form a vital component of the BM niche, contributing to multilineage potential, immunomodulatory functions, and aiding in maintaining HSC quiescence [6].

AML-BM-MSC are reported to contribute to disease progression, chemoresistance, and restricted apoptosis in AML blasts via cell–cell interaction, cytokine–receptor interactions, and released exosomes in the microenvironment [7–9]. On the other hand, a contradictory role of BM-MSC includes tumor growth inhibition [10, 11]. Lopes et al*.* [12] demonstrated altered cytokine patterns in AML patients, including elevated levels of chemokines CXCL12, inflammatory cytokines IL-1*β*, IL-6, etc. Conditioned medium (CM) derived from MSC has been shown to exert dynamic effects on cancer cell growth. MSC-CM from a healthy individual BM promoted the proliferation of multiple myeloma cell lines [13]. Human umbilical cord (UC) MSC-CM in association with tamoxifen was reported as a potential therapeutic option in modulating tamoxifen chemosensitivity by regulating the BRCA1 gene in breast cancer [14]. Chronic inflammation is a fundamentally contributing factor in cancer initiation and progression [39]. One of the signaling cascades contributing to inflammation is the nuclear factor of *κ*-light chain of enhancer of activated-B cells (NF-*κ*B) that results in transcription and release of key pro-inflammatory cytokines such as IL-1 and IL-6. which promote tumorigenesis [15, 16]. Furthermore, multiple downstream pathways following NF-*κ*B result in the activation of one of the cytosolic sensory proteins, thus causing assembly and activation of a multiprotein complex called the inflammasome, which in turn causes activation of caspase-1 and further cleavage of pro-inflammatory cytokines IL-1*β*, IL-18 and subsequent release into the intercellular space [17]. This release contributes to further signaling and activation of cytokines and chemokines, resulting in enhanced inflammation.

How AML patient-derived BM-MSC-CM governs AML cell fate remains unanswered. Therefore, in this study, we cultured AML patient-derived bone marrow MSC and characterized them for alterations at the level of ultrastructural morphology, cell cycle, surface marker expression, and gene expression profiles. Further, we also evaluated the role of AML-BM-MSC and PD-MSC-CM in governing AML cell fate in in vitro cultures as well as leukemia tumor growth xenograft in an immunodeficient mouse model.

## Materials and methods

### Study subjects

The study included newly diagnosed AML patients, and 3–5 mL BM samples were collected from patients at diagnosis (*n* = 6) attending the outpatient department of ACTREC-TMC. BM (*n* = 2) from Early T-cell precursor acute lymphoblastic leukemia (ETP-ALL, precursor myeloid/lymphoid leukemia) patients were also included as controls. BM samples from Non-Hodgkin lymphoma (NHL) patients (*n* = 2) having BM devoid of blasts (uninvolved BM—Un-BM) were included as a control as per IEC recommendation. Healthy individuals (HI, *n* = 5) peripheral blood (PB) plasma was used as a control for soluble factor studies. Demographic data for patients and healthy controls included in the study are shown in Supplementary Table ST1. All BM samples were collected after written informed consent from patients at Tata Memorial Hospital and ACTREC-TMC, and the collection procedure was in accordance with the approved protocols of the human ethics committee of Tata Memorial Centre (IEC-III approved studies #100 and #900561).

### Cell lines and reagents

The OCI-AML2 cell line was kindly provided by Dr Syed Hasan (Hasan Lab, ACTREC-TMC). HL-60, KG-1, and K-562 cell lines were procured from the National Cancer Institute, USA. Umbilical cord Wharton jelly-derived-MSC (UC-MSC) were procured from OCT Therapies and Research Pvt Ltd, Mumbai, and were included as a control for mechanistic studies. Normal-Bone marrow-mesenchymal stem cells (N-BM-MSC) were commercially procured (Hi-Media Laboratories Pvt Ltd, India), as a comparison control for patient-derived-mesenchymal stem (PD-MSC-CM) experiments. The culture conditions for all the cells are provided in Supplementary Methods. Details of the reagents are provided in Supplementary Table ST2.

### Sample processing, enrichment and maintenance of BM-MSC

BM/PB samples were processed as described previously [18], centrifuged, and plasma was collected, aliquoted, and stored at − 80 °C for evaluating soluble marker expression. BM samples were processed to obtain enriched MSC from AML/ETP-ALL/lymphoma patients as described previously [19]. MSC could be successfully cultured up to passage P3/P5 and further characterized based on the criteria devised by the International Society for Cellular Therapy (ISCT) [20]. Detailed methodology is provided in the Supplementary Methods.

### Collection and storage of PD-MSC-CM from cultured MSC

Conditioned medium (CM) was collected from BM-MSC (AML and ETP-ALL patients) after attaining a confluence of 70–80%. The collected CM was centrifuged, and cell-free CM was stored at – 80 °C for all PD-MSC-CM experiments.

### Characterization of AML-BM-MSC

#### Phenotyping by flow cytometry

Expression of surface markers on MSC was evaluated by multicolor immunophenotyping as reported previously, [19]. In brief, isolated and cultured AML-BM-MSC were trypsinized, fixed with 1% paraformaldehyde (PFA), stained with conjugated antihuman antibodies, and acquired using FACS Aria III, Flow cytometer (Becton Dickinson, USA). Detailed methodology is provided in the Supplementary Methods.

#### Morphological characterization of cultured AML-BM-MSC

Ultrastructural morphology in MSC was assessed using a transmission electron microscope (TEM, JEOL1400 plus, Japan) as per the protocol described by [19, 21, 22]. Briefly, AML-BM-MSC and UC-MSC were grown in monolayers up to 70–80% confluence. To mimic the altered MSC phenotype in AML-BM de novo, we created a model by co-culturing UC-MSC with the OCI-AML2 cell line for 24 h, and the resulting morphological alterations were evaluated by TEM. MSC were analyzed for mitochondrial and intracellular vesicle numbers using i-TEM Analysis Software (Olympus, Germany). Detailed methodology is provided in the Supplementary Methods.

#### Lineage differentiation potential of AML-BM-MSC

Multilineage differentiation potential of AML-BM-MSC was evaluated for osteogenic and adipogenic lineages as described previously [19]. Briefly, AML-BM-MSC were cultured in MesenCult™ basal media supplemented with osteogenic and adipogenic stimulatory factors and incubated in a humidified CO_2_ incubator at 37 °C. The experiment was conducted using two biological replicates. Detailed methodology is provided in the Supplementary Methods.

#### Comparison of gene expression profiles of AML-BM-MSC and Un-BM-MSC

AML-BM-MSC (*n* = 2) were evaluated for their differential gene expression (DGE) in comparison to Un-BM-MSC (*n* = 2) by microarray hybridization. The differential gene expression analysis was conducted using the Limma-voom package in R software. Further, a ranked list of the deregulated genes was taken up for gene set enrichment analysis (GSEA) using the WebGestalt online tool (https://www.webgestalt.org/) [23]. Detailed methodology is provided in the Supplementary Methods.

### Validation of expression of inflammasome pathway gene in AML-BM-MSC by quantitative real-time polymerase chain reaction

AML-BM-MSC (*n* = 2) were evaluated for the differential expression of inflammasome pathway genes, viz. NLRP3, IL-18, and Caspase-1 in comparison with UC-MSC by quantitative real-time polymerase chain reaction (*q*-PCR). 18S rRNA was used as a housekeeping gene. In brief, cultured AML-BM-MSC and healthy UC-MSC were fixed in TRIzol and stored at − 80 °C, and processed for RNA extraction and cDNA synthesis. Detailed methodology and primer sequences are provided in the Supplementary Methods and Supplementary Table ST3.

### Characterization of MSC-CM derived from AML patients

The medium composition of PD-MSC-CM from AML patients (*n* = 5) was analyzed for cytokine secretion using AimPlex® Bead-Based Multiplex Immunoassay for growth factors, as per the manufacturer’s instructions. Briefly, PD-MSC-CM samples were incubated with soluble cytokine/growth factor-specific beads, washed, stained with their respective secondary antibodies, and the concentration of each growth factor was determined by Flow cytometric analysis. Detailed methods are provided in Supplementary Methods.

### Evaluation of inflammatory cytokine profile in AML-BM-MSC-CM and co-culture supernatants

Inflammatory cytokine profiling of AML-BM-MSC: OCI-AML2 co-culture supernatants were determined by Cytokine bead array (CBA) as described [18]. Briefly, OCI-AML2 cells were cultured either as single cells or with AML-BM-MSC (cell–cell contact) for 48 h. Culture supernatants were collected, centrifuged, and evaluated for an inflammatory cytokine profile. CBA analysis was conducted using two experimental replicates for every PD-MSC-CM sample. Detailed methodology is provided in the Supplementary Methods.

### Cytocompatibility of PD-MSC-CM using healthy peripheral blood lymphocytes

The biocompatibility of PD-MSC-CM (*n* = 5) and N-BM-MSC (*n* = 1) was tested by assessing their effect on the metabolic activity of PBL. PBL from healthy individuals (*n* = 3) were seeded in a 96-well plate (10,000 cells/well) with PD-MSC-CM (10%). Cells were incubated at 37 °C in the presence of 5% CO2 for 48 h. Following incubation, CCK-8 reagent was added to each well and incubated for 4 h at 37 °C. Absorbance was recorded using an Agilent Biotek Elx8 microplate colorimetry reader (Absorbance 450 nm). The assay was conducted as three technical replicates. Data were represented as mean % metabolic activity and standard error.

### Effect of PD-MSC-CM on immune cell-mediated cytotoxicity against NK-sensitive leukemia target 162 (K-562)

The immunomodulatory function of PD-MSC-CM was evaluated by modulating immune cell-mediated cytotoxicity against the human leukemia cell line K-562, a known target for natural killer cell-mediated cytotoxicity. Freshly isolated PBL from healthy individuals (*n* = 2) were co-cultured with target cells labeled with Calcein-AM (Thermo Fisher Scientific) at 2 μM concentration. PBL were co-cultured with K-562 at E:T ratios of 5:1, 10:1, 20:1 in the presence of PD-MSC-CM (10 and 50% concentration). The experiment was conducted in three technical replicates. After incubation for 4 h, cell-free supernatants were collected and analyzed on a Biotek Cytation microplate spectrofluorometer (excitation filter: 488 nm; band-pass filter: 523 nm) (Agilent Technology, BioTek Elx8 microplate reader, USA, Software: Agilent BioTek Gen5 software USA). The percent lysis or cytotoxicity was calculated to assess cell-mediated cytotoxicity.

### Effect of AML-BM-MSC and PD-MSC-CM on AML cell growth potential by in vitro assays

The effect of AML-BM-MSC (*n* = 2) and PD-MSC-CM (*n* = 4) on OCI-AML2 cell growth was evaluated by 3-(4,5-dimethylthiazol-2-yl)-2,5-diphenyltetrazolium bromide (MTT) and sulforhodamine B (SRB) staining assay as described by [19]. Briefly, OCI-AML2 cells were cultured with AML-BM-MSC and PD-MSC-CM for 48 h in the presence of cytarabine (1 and 2 µM). For the MTT experiment, adriamycin (ADR, 1 µM) was used as a positive control. Co-cultured OCI-AML2 cells were collected, suspended in 96-well plates, and stained with MTT. Absorbance was recorded at 540 nm to determine % viability, whereas OCI-AML2 cultured with PD-MSC-CM in the presence of cytarabine (1 µM) were stained with SRB. Absorbance was recorded at 565 nm, and percent growth was calculated. Detailed methodology is provided in Supplementary Methods.

### Co-culture interactions between MSC and AML cells

#### 2.12.1. Transwell migration assay

Cell–cell interaction between AML-BM-MSC and OCI-AML2 cells was evaluated by transwell migration assay as described previously [5]. Briefly, AML-BM-MSC were cultured in the lower chambers in the presence of MEM-α (2% FBS), whereas OCI-AML2 (MEM-α without FBS) was seeded in the upper chambers. Cultures were incubated for 24 h, and OCI-AML2 cells in the lower chamber were enumerated by counting the number of cells per section for seven sections. The experiment was conducted as two technical replicates. Detailed methodology is provided in Supplementary Methods.

#### Live cell monitoring using a laser confocal microscope

To validate the findings, a real-time imaging experiment was conducted using a 3i spinning disk confocal microscope at 100 × magnification.

#### Mitochondrial transfer study

Similarly, the mitochondrial transfer ability of MSC to OCI-AML2 cells was studied using the Incucyte S3 live-cell analysis system at 20 × magnification, following a modified protocol described in [24]. For both experiments, MSC and OCI-AML2 cells were stained and co-cultured for 1–4 h, after which live-cell imaging was performed. Detailed methodology is provided in the Supplementary Methods.

#### Evaluation of the effect of PD-MSC-CM on AML cell mitochondrial membrane potential in AML cells

Alterations in mitochondrial membrane potential in OCI-AML2 cells cultured with PD-MSC-CM were performed as per the protocol described previously [25]. Briefly, OCI-AML2 cells were cultured with PD-MSC-CM (100%) in the presence/absence of cytarabine. Treated cells were stained with the JC-1 dye and acquired using a flow cytometer (Attune NXT, Thermo Fisher Scientific, USA). Data were analyzed using FlowJo software (BD Biosciences, USA). Detailed methodology is provided in the Supplementary Methods.

#### Evaluation of the effect of PD-MSC-CM on inflammasome protein expression in AML cells

The effect of PD-MSC-CM (50%) on NLRP3 protein expression was evaluated by immunofluorescence assay. Briefly, OCI-AML2 cells were stimulated with LPS and ATP to induce inflammasome activation in the presence of PD-MSC-CM (*n* = 4) for 24 h. Cells treated with MCC950 (5 µM) were included as a positive control for NLRP3 inhibition. The resultant cells were fixed and stained with antibodies against NLRP3. The detailed methodology is described in the Supplementary Methods.

#### Evaluation of the effect of AML-BM-MSC and PD-MSC-CM on AML cell cycle phases in AML cells

The effect of AML-BM-MSC and PD-MSC-CM (100%) on OCI-AML2 cell cycle phases was evaluated as per the protocol described [26]. Briefly, OCI-AML2 cells were incubated in the presence of PD-MSC-CM (non-cellular contact)/AML-BM-MSC (cell–cell contact) with/without cytarabine (1 and 2 µM) for 48 h. Incubated OCI-AML2 cells were collected, fixed, and stained with propidium iodide and acquired on a flow cytometer (Attune NxT, Thermo Fisher Scientific, USA). Data were analyzed using the ModFit software (Version 5.0, Verity Software House, USA). Detailed methodology is provided in the Supplementary File.

#### To study the effect of PD-MSC-CM on leukemia tumor growth in an immunodeficient mice model

The tumor-reduction potential of PD-MSC-CM (100%) was evaluated in a leukemia xenograft model in immunodeficient mice, as described previously [26]. All procedures involving mice were performed according to protocols approved by the Institutional Animal Ethics Committee, ACTREC, Tata Memorial Centre, Navi Mumbai (IAEC Approval # 1/2015 and #43/2022), and in accordance with CCSEA guidelines (Registration Number: 65/GO/ReBiBt/S/99/CCSEA). Animals received humane care, and all efforts were undertaken to minimize animal suffering before and during the experiments. For the in vivo experiment, the ethical guideline was to euthanize mice when the tumors reach 2000 mm^3^ in volume (10% of body weight), or as soon as tumors show signs of necrosis, ulceration, or bleeding. However, in our study, this endpoint was not reached. In all in vivo experiments, mice were killed by lethal exposure to CO_2_ followed by cervical dislocation.

Non-obese diabetic (NOD-SCID) mice, 6–8-week-old male mice weighing 20–25 g, bred in the Laboratory Animal Facility at ACTREC, were used for the study. An exploratory in vivo efficacy study using ETP-ALL BM-MSC-CM treatment in the KG-1 leukemia xenograft model was conducted. Mice were randomized in groups (*n* = 2/per group), viz. (A) tumor-bearing control, (B) positive control ADR treated, and (C) ETP-ALL-MSC-CM-treated group. The study period was 31 days.

In the main experiment on K-562 xenograft tumors, mice (*n* = 4/group) were randomized into four different groups, viz. (A) tumor-bearing control, (B) positive control 5-FU treated, (C) N-BM-MSC-CM-treated, and (D) PD-MSC-CM-03-treated group. The dosing protocol for 5-FU was 5 mg/kg via the intraperitoneal (i.p.) route up to the ninth day. The protocol for the CM treatment groups (C) and (D) was 200 µL CM/mouse i.p. thrice a week for 4 weeks. Mice were monitored every fourth day for body weight, tumor volume, and mortality over 30 days. Tumor volume was measured using Vernier Caliper. Mice were sacrificed on the 31st day, and tumors were collected and fixed for further analysis. Relative tumor volume was calculated as per the protocol described [27]. Detailed analysis methodology is provided in the Supplementary File.

#### Validation of inflammasome marker expression by immunohistochemistry

Evaluation of inflammasome markers in AML-BM sections and mice xenograft tumors was conducted by immunohistochemistry. Briefly, FFPE sections were processed for deparaffinization, rehydration, peroxidase blocking, and staining for inflammasome markers NLRP3, IL-1*β*, and Caspase-1. The following day, sections were stained with HRP-conjugated secondary antibodies and substrate buffer. Slides were stained with nuclear stain hematoxylin. Images were captured using Zeiss Axio Imager Z1 and analyzed using microscope-associated analysis software (ZEN 3.0 (blue edition), Carl Zeiss Microscopy GmbH, 2019). Detailed methodology is provided in the Supplementary Methods.

#### Immunoassay to estimate the expression of soluble IL-18 levels in AML plasma samples at diagnosis

Soluble IL-18 expression was assessed in AML BM plasma (*n* = 4) at diagnosis, compared with healthy PB plasma (*n* = 5), using a conventional sandwich ELISA as described previously [28]. Briefly, plasma samples were added to antibody-coated wells and incubated with a detection antibody, enzyme, and TMB substrate. Recombinant cytokine standards were used. Only correlation coefficients (*r*^2^) > 0.9 were accepted for interpolating unknown values. Absorbance was recorded at 450 nm. Detailed methodology is provided in the Supplementary Methods.

#### Statistical analysis

The Shapiro–Wilk normality test was used to determine the normality of the data. Data are presented as mean ± SD for normally distributed variables and as median (IQR) for non-normally distributed variables. An unpaired *t*-test was used for the comparisons between two independent groups. One-way ANOVA followed by a post hoc (multiple comparison) test was used to check the differences in mean values among more than two independent groups. A *p*-value ≤ 0.05 was considered statistically significant. Statistical analyses were performed using GraphPad Prism version 8.0 (GraphPad Software, San Diego, CA, USA) and IBM SPSS Statistics version 25.0 (IBM Corp., Armonk, NY, USA).

## Results

### BM stroma in AML patients harbor MSC with distinct features

**Fig. 1:**
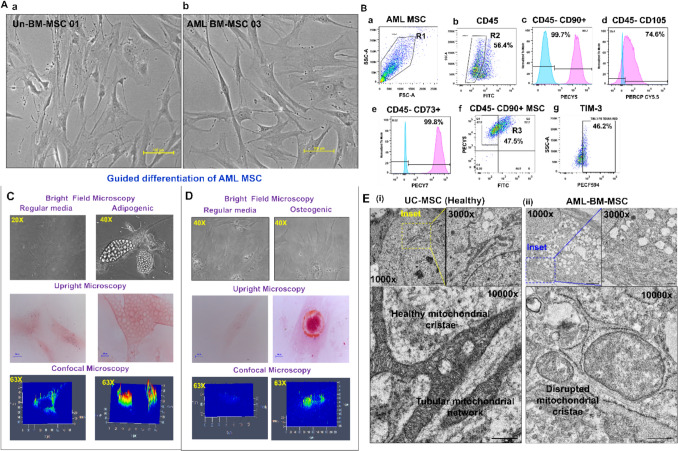
Isolation and characterization of AML patient-derived BM-MSC. **A** Bright field microscopic images of **A-a** Un-BM-MSC and **A-b** AML-BM-MSC displaying elongated fibroblastoid morphology. **B** Flow cytometric analysis of standard surface markers in AML-BM-MSC at passage P3. **B**-**a** MSC are gated on forward scatter and side scatter (*R*1). **B**-**b** demonstrates the *R*1 cells gated for the CD45-negative population (*R*2). Further expression of MSC-specific markers CD90 **B**-**c**, CD105 **B**-**d**, and CD73 **B**-**e** on the *R*2 gated population is shown, where negative controls are represented by blue histograms and test marker expression by pink histograms. **B**-**f** demonstrates a dot plot of AML-MSC gated from the R1 population to obtain the *R*3 population having CD45-CD90 + expression. This *R*3 gated population was analyzed for TIM-3 (**B**-**g**) expression. **C** and **D** Lineage differentiation potential of AML-BM-MSC into Adipogenic and Osteogenic lineages is shown as bright field (20 × and 40x) and upright microscopic images of Oil Red O staining for adipogenic potential and Alizarin Red staining for osteogenic potential as well as 3D interactive intensity plots of positively stained images. **E** Images (i) and (ii) are shown at 1000X, 3000X, and 10000X magnification. The yellow (−) box in image (i) and the blue (−) box in image (ii) are in focus. A scale bar is depicted at the bottom right of Panels A, C, D, and E

Mesenchymal stem cells were successfully isolated and cultured from 6 of 10 AML, 1 of 3 ETP-ALL, and 2 lymphoma patients (Un-BM). AML-BM-MSC displayed a heterogeneous, elongated, fibroblastoid morphology (Fig. 1A, Supplementary Fig. S1). AML-BM-MSC demonstrated variable expression of CD90hi, CD105hi, CD73hi, and CD45lo, and further expressed other immune markers, viz. toll-like receptors TLR3 and TLR4, the immune checkpoint marker TIM-3, and immunosuppressive adenosine receptors A2AR and A2BR (Fig. 1B and Supplementary Fig. S2). AML-BM-MSC demonstrated capacity to differentiate into adipogenic (Fig. 1C) as well as osteogenic lineages (Fig. 1D). On comparison at the ultrastructural level, AML-BM-MSC exhibited dense cytoplasm compared to UC-MSC (Supplementary Fig. S3A). AML-BM-MSC demonstrated increased intracellular vesicles localized at the cellular periphery, whereas UC-MSC demonstrated a lower number of cellular vesicles (***p* = 0.004**, 66.6 ± 13.54) as evaluated by TEM analysis (Supplementary Fig. S3A, S3B). Furthermore, AML-BM-MSC revealed an altered mitochondrial shape in comparison to UC-MSC (Fig. 1E, Supplementary Fig. S3A).

To further investigate and validate these findings, we mimicked an in vitro assay in which UC-MSC co-cultured with OCI-AML2 cells for 24 h were evaluated for morphological alterations by transmission electron microscopy (now designated as UC-MSC*). We observed increased mitochondrial number (***p* = 0.024**, 17.8 ± 6.8), and vesicle number per section (***p* = 0.004**, 137.0 ± 34.53, Supplementary Fig. S4A, S4B) in UC-MSC* compared to control UC-MSC. In addition, endoplasmic reticulum (ER) dilation was also observed in co-cultured UC-MSC* compared to control UC-MSC represented by increased ER lumen width (***p* < 0.0001**, 26.24 ± 2.52, Supplementary Fig. S4C).

### Differential gene expression profiling in AML-BM-MSC revealed pathway dysregulation compared to Un-BM-MSC

**Fig.2:**
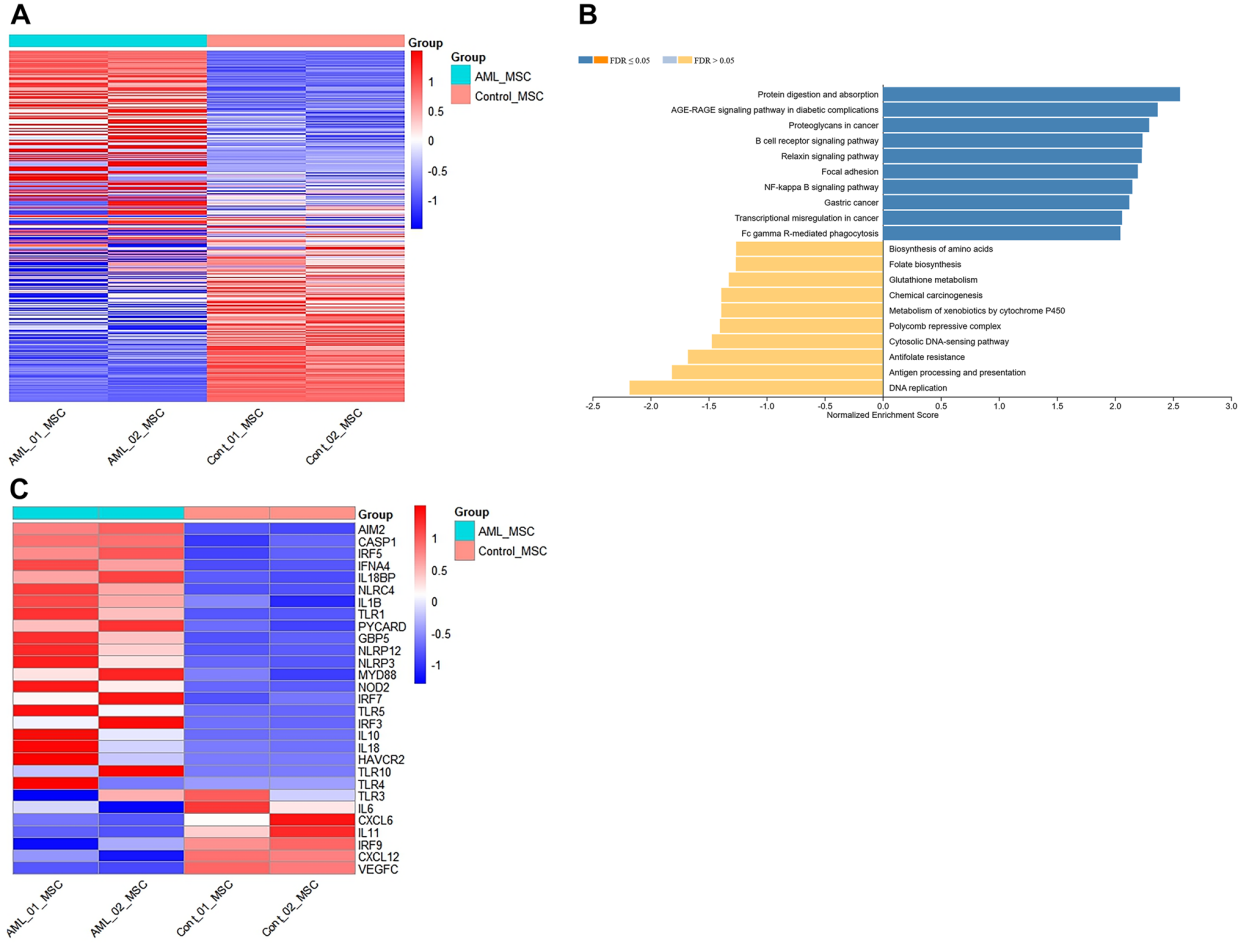
Differential gene expression in AML-BM-MSC at diagnosis. Panel **A** depicts the heatmap of normalized counts showing upregulated genes (red) and downregulated genes (blue) across AML_MSC control vs control (Un-BM) MSC group analysis using Limma-voom. Panel **B** demonstrates gene set enrichment analysis (GSEA) in AML-BM-MSC (*n* = 2) compared to Un-BM-MSC (*n* = 2), represented as a normalized enrichment score (NES) plot with FDR values of ≤ 0.05 is considered statistically significant. Panel **C** shows a heatmap of key inflammatory pathway genes that are differentially expressed in AML_MSC vs. Control (Un-BM) MSC

The DGE analysis using Limma-voom showed significant deregulation of 2926 genes between AML-BM-MSC and Control (Un-BM-MSC; Supplementary Fig. S5). Among these 1175 genes, 1175 were upregulated and 1751 were downregulated, as shown in the heatmap (Fig. 2A). Gene set enrichment analysis (GSEA) identified significantly enriched pathways as upregulated, as visualized in the NES plot. Notable pathways, viz. protein digestion and absorption, focal adhesion, proteoglycans in cancer, and NF-*κ*B showed significant enrichment, with FDR values of ≤ **0.05** as evidenced by positive NES (Fig. 2B). Additionally, over-representation analysis (ORA) identified ten significantly enriched pathways. Other statistically significant pathways also showed enrichment scores greater than 2. Gene ontology (biological process) identified the top ten enriched pathways, as shown in Supplementary Fig. S5, with FDR < **0.05**. Furthermore, we explored deregulated genes across various inflammatory pathways and identified upregulated genes in the inflammasome pathway, viz. AIM-2, CASP-1, IL-1*β*, IL-18, NLRC4, PYCARD, NLRP3, and NLRP12 (Fig. 2C). Further, we validated expression of inflammasome pathway gene NLRP3, IL-18, and CASP-1 in AML-BM-MSC (*n* = 2) in comparison to healthy UC-MSC by qPCR (Supplementary Fig. S6). Interestingly, these observations were corroborated by clinical AML BM plasma samples (*n* = 4, 542.3 ± 92.43) that also exhibited significantly increased levels of sIL-18 (*p* = 0.01) compared to healthy plasma (*n* = 5, 210.9 ± 44.86) (Supplementary Fig. S7).

### Dynamics of intercellular interactions among AML-BM-MSC and OCI-AML2 underscore the protective role of BM-MSC in the presence of drug treatment

**Fig.3:**
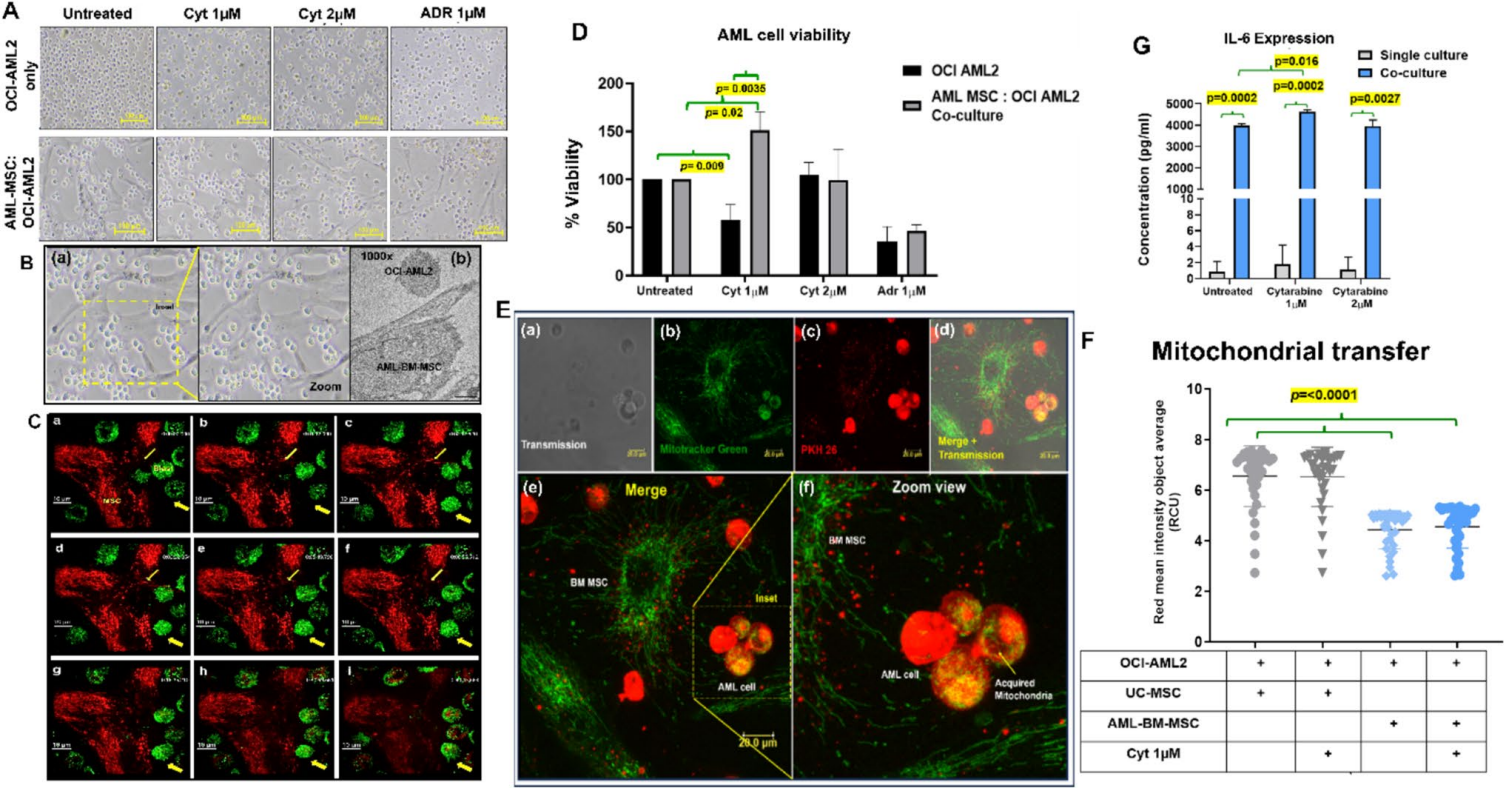
Interaction between AML-BM-MSC and leukemia cells in in vitro assays. **A** Bright-field microscopic images of single and co-cultures of OCI-AML2 and AML-BM-MSC. **B** Enlarged insets of **a** bright field images of co-cultures at 100 µm and **b** an electron microscopic image demonstrating OCI-AML2 adherence to AML-BM-MSC at 1000x. **C** Live cell imaging of OCI-AML2 (PKH-67) recruitment to AML-BM-MSC (Mito tracker CMX ROS) by confocal imaging at 10 µm. **D** Percent viability of OCI-AML2 cells by MTT assay in single and co-cultures. Adriamycin (ADR; 1 µM) was used as a positive control. The experiment was conducted in two biological replicates. **E** Representative confocal microscopic image of mitochondrial transfer from AML-BM-MSC (Mito tracker green FM) and leukemia cell (HL-60) (PKH-26) cells. The panels exhibit **a** only transmission, **b** only green, **c** only red-stained images, and **d** merged. **e** Demonstrates the kinetics of bidirectional transfer of mitochondria (green) to leukemic blasts (red) and transfer of vesicles (red) pinched from leukemic blasts. **f** The panel shows an enlarged version of the bi-directional transfer. The red-stained AML cells that accommodate mitochondria from AML-BM-MSC appear yellow due to co-localization, as demonstrated in the inset figure. Images were captured at 20 µm. **F** Quantification of transferred mitochondria from stained MSC (both AML-BM-MSC and Healthy UC-MSC- Mito tracker CMX Ros-red) to OCI-AML2 (PKH67 green) cells by evaluating red mean square intensity object average values after co-culture for 4 h. **G** Inflammatory cytokine expression in single and co-culture supernatants in the presence and absence of cytarabine. Each experiment was conducted in three technical replicates. Significant *p*-values are highlighted and accompanied by the Mean ± SEM values

AML-BM-MSC and OCI-AML2 co-cultures demonstrated the recruitment of leukemic cells (HL-60 and OCI-AML2) to the surface of AML-BM-MSC (Fig. 3A, 3B, Supplementary Video SV1, and Supplementary Fig. S8–S10). Live-cell confocal microscopy revealed that OCI-AML2 cells moved toward AML-BM-MSC. This recruitment occurred within a time frame of 5–30 min after seeding OCI-AML2 cells (Fig. 3C). Transwell migration assay demonstrated active migration (*p* **<** **0.0001**) of OCI-AML2 cells to AML-BM-MSC (Cell count 354.4 ± 13) in comparison to wells containing only OCI-AML2 (cell count 94.57 ± 24). Cell recruiting effect was further enhanced in response to cellular stress induced by Cytarabine (1 µM) treatment (***p* = 0.009**), as shown in Supplementary Fig. S11. Evaluation of Cytarabine-induced growth inhibition of OCI-AML2 in the presence of AML-BM-MSC was conducted using the MTT assay. It was observed that Cytarabine alone caused reduced growth of OCI-AML2 cells compared to control (***p* = 0.009**, 56.5 ± 41.4, Fig. 3D). However, this effect of cytarabine was reversed when OCI-AML2 were co-cultured with AML-BM-MSC (***p* = 0.02**, 150.9 ± − 50.92). Data were validated by comparing cell viability (Supplementary Fig. S12). It was observed that the effect of Cytarabine on S-phase accumulation of OCI-AML2 had a marginal impact on co-culture (Supplementary Fig. S13).

At the cellular level, it was observed that co-culture of AML-BM-MSC leukemia cells (HL-60) demonstrated mitochondrial transfer, which was observed at 2 h of co-culture (Fig. 3E). Live tracking of this transfer demonstrated initiation of mitochondrial transfer from AML-BM-MSC to OCI-AML2 cells as early as 30 min of co-culture initiation (Fig. 3C, and Supplementary Video SV2). We compared the mitochondrial transfer ability of MSC (healthy UC-MSC and AML-BM-MSC) using live-cell monitoring. We observed that AML-BM-MSC exhibited a significantly reduced transfer ability compared to healthy UC-MSC (***p* < 0.0001, − 2.120 ± 0.23**, Fig. 3F). However, both MSC (healthy UC-MSC and AML-BM-MSC) blocked the effect of cytarabine-induced reduction of mitochondrial transfer ability.

Inflammatory cytokine profiling of culture supernatants demonstrated increased sIL-6 levels in co-culture supernatants compared to only OCI-AML2 cultures (***p* = 0.0002**, 3995 ± 52.38 for untreated controls, ***p* = 0.0002** 4619 ± 61.66 for Cytarabine (1 µM), and ***p* = 0.0027,** 3949 ± 206.6 for Cytarabine (2 µM) treatment, respectively) as shown in Fig. 3G. In addition, it was noted that cytarabine (1 µM) treatment further enhanced sIL-6 secretion (***p* = 0.016, 625.6 ± 80.89**) in co-cultures (Fig. 3G). Similar results were obtained with sIL-8 in culture supernatants (Supplementary Fig. S14).

### PD-BM-MSC-CM displays leukemia reduction in pre-clinical in vitro and in vivo models

#### PD-MSC-CM composition

**Fig. 4:**
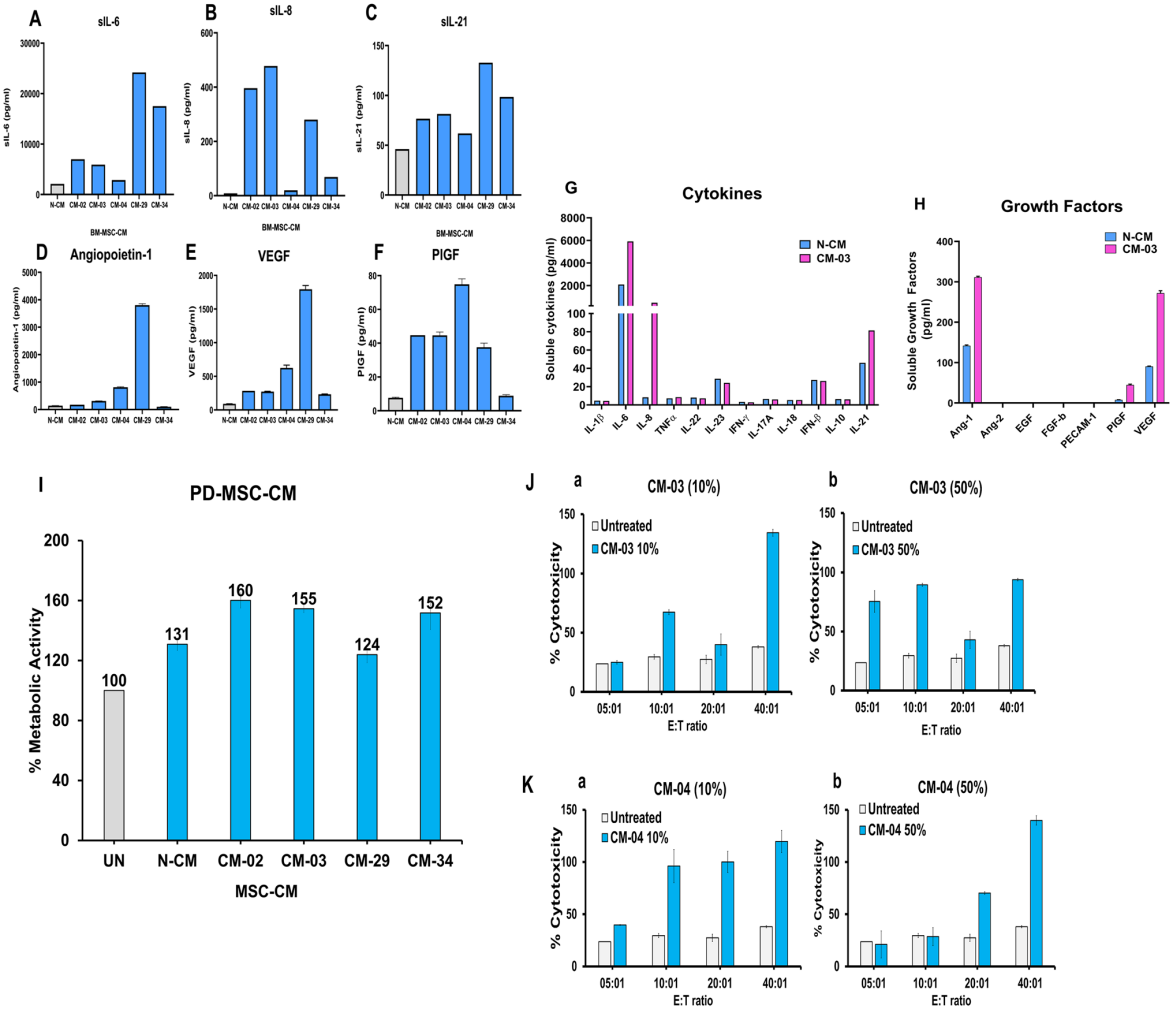
Composition and immunomodulatory properties of PD-MSC-CM. **A**–**C** Bar graphs depicting the quantification in pg/ml of different cytokines in PD-MSC-CM (*n* = 5), **A** sIL-6, **B** sIL-8, and **C** sIL-21, and soluble growth factors **D** Angiopoietin-1, **E** VEGF, and **F** PlGF in PD-MSC-CM. **G** and **H** Bar graphs showing the overall soluble cytokine and growth factor profiles of PD-MSC-CM-03 in comparison to N-BM-MSC-CM. **I** Bar graphs showing the effect of PD-MSC-CM (*n* = 4) on % metabolic activity of healthy PBLs. Panels demonstrate the effect of PD-MSC-CM 03 J and CM-04 K on Natural Killer cell-mediated % cytotoxicity in K-562 target cells (at different E: T ratios) at a concentration of 10% (**I**-**a** and **J**-**a**) and 50% (**I**-**b** and **J**-**b**). All experiments were conducted in three technical replicates

The soluble marker profile of PD-MSC-CM was comparable to N-MSC-CM (Fig. 4A–F). PD-MSC-CM-03 was found to exhibit distinct levels of immune markers sIL-6, sIL-8, sIL-23, sIFN-*β*, and sIL-21 (Fig. 4G). The growth factors found were angiopoietin-1, PlGF, and VEGF (Fig. 4H). Other CM followed a similar trend; however, the levels differed (Supplementary Fig. S15 and S16, and Supplementary Tables ST4 and ST5).

#### Immunomodulatory properties

PD-MSC-CM did not hamper the metabolic activity of healthy peripheral blood cells (Fig. 4I), indicating cytocompatibility with immune cell activity. Further, it was found to enhance immune-mediated kill of NK-sensitive leukemia target K-562 cells (Fig. 4J and 4K). The cell cytotoxicity effect was found to be dose-dependent at 10% for both and 50% in PD-MSC-CM-04. At higher concentrations, PD-MSC-CM was directly cytotoxic to leukemia cells, as indicated by spontaneous release values (data not shown). Hence, PD-MSC-CM-03 was chosen for the in vivo efficacy assay.

#### In vitro anti-leukemia activity and mechanism enhancing sensitivity to chemotherapeutic drug Cytarabine

**Fig. 5:**
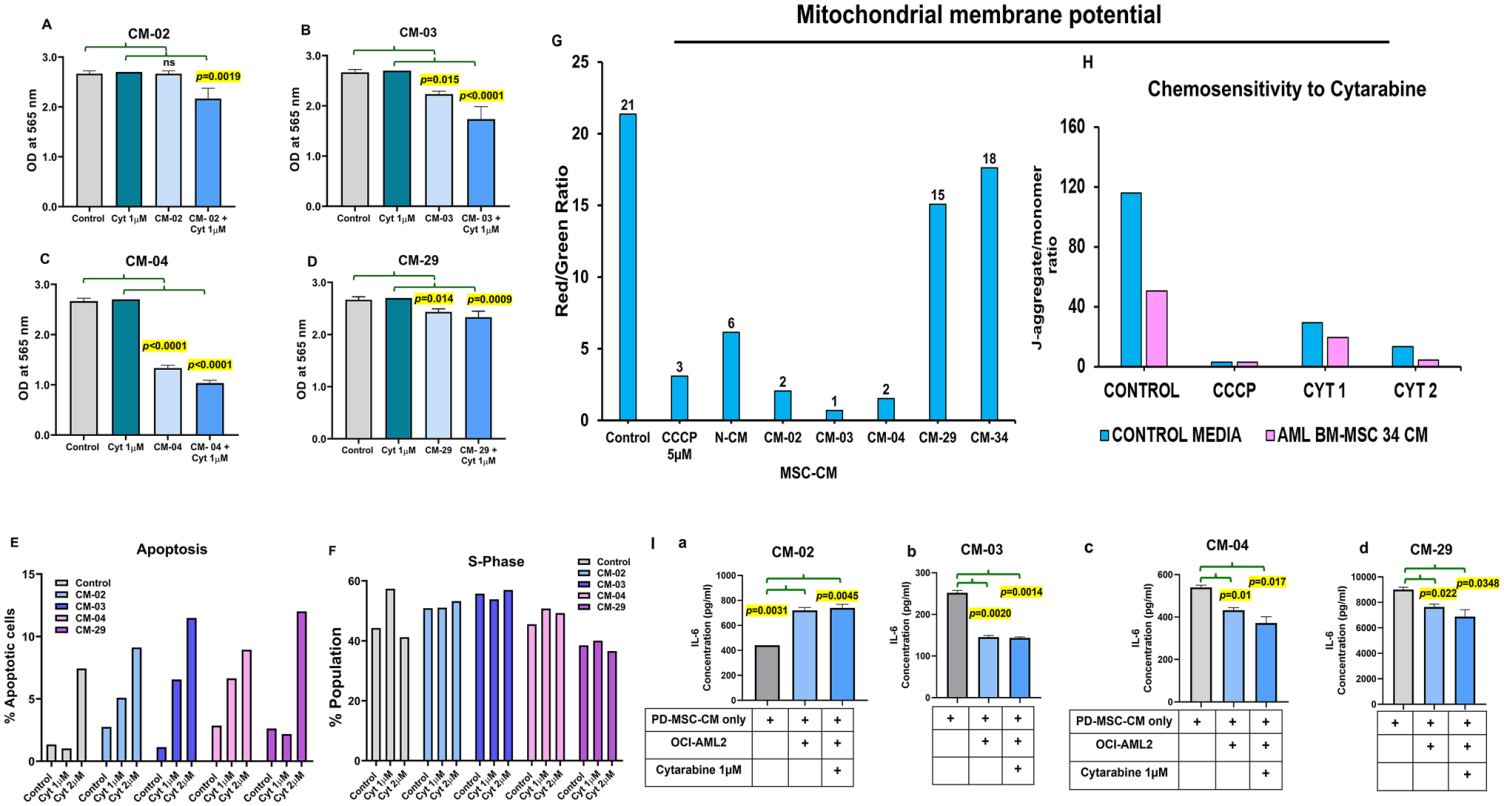
Anti-leukemic effect of PD-MSC-CM and enhancing chemosensitivity to Cytarabine. Bar graph demonstrating growth inhibitory effect of PD-MSC-CM **A** CM-02, **B** CM-03, **C** CM-04, and **D** CM-29 on OCI-AML2 in the presence/absence of Cytarabine treatment (1 µM) by SRB assay. The experiment was conducted in three technical replicates. Cell cycle analysis showing **E** % Apoptotic cells and **F** % S-phase population of OCI-AML2 treated with PD-MSC-CM in the presence of Cytarabine. **G** Effect of PD-MSC-CM (*n* = 5) on Mitochondrial membrane potential of OCI-AML2. **H** Bar graph showing synergistic effect of PD-MSC-CM-34 and Cytarabine on mitochondrial membrane potential of OCI-AML2. **I** sIL-6 cytokine secretion alteration in OCI-AML2 cell supernatants treated with PD-MSC-CM (*n* = 4)

The PD-MSC-CM demonstrated a direct growth inhibitory effect on OCI-AML2 cells, and the effect was found to be further augmented in the presence of cytarabine (Fig. 5A–D). One-way ANOVA analysis demonstrated that the growth inhibition was statistically significant (CM-03 ***p* = 0.015**; CM-04 ***p* < 0.0001**; and CM-29 ***p* = 0.014**) compared to untreated controls. This data indicated that PD-MSC-CM distinctly enhances chemosensitivity of OCI-AML2 to cytarabine.

Further, we observed that the synergistic inhibitory effect of PD-MSC-CM on cytarabine-treated OCI-AML2 cells was mediated by its marked effects on apoptosis (Fig. 5E) and the S-phase cell population (Fig. 5F). Apoptosis induction was found to be dose-dependent (Fig. 5E). Next, we observed that PD-MSC-CM (*n* = 5) also acted by causing mitochondrial depolarization in OCI-AML2 cells (Fig. 5G and Supplementary Fig. S17). The PD-MSC-CM-03 demonstrated an enhanced effect compared to N-CM and was therefore selected for the in vivo efficacy experiment. It was interesting to note that the chemosensitivity-enhancing effect of PD-MSC-CM toward cytarabine-induced growth inhibition (Fig. 5B) could be attributed to mitochondrial membrane depolarization in OCI-AML2 cells (Fig. 5H).

Further, the growth inhibitory effect of PD-MSC-CM was found to be linked to the reduction of sIL-6 (CM-03 ***p* = 0.002**; CM-04 ***p* = 0.01**; and CM-29 ***p* = 0.022**) by OCI-AML2 cells (Fig. 5I). The MSC-supported survival and increase in sIL-6 by OCI-AML2 in the presence of cytarabine treatment (Fig. 3G) were found to be curbed in the presence of PD-MSC-CM (Fig. 5I).

#### In vitro anti-leukemia activity through NLRP3 pathway regulation

To elucidate the mechanistic action of PD-MSC-CM on leukemia growth reduction, the OCI-AML2 inflammasome activation model was treated with NLRP3-specific inhibitor MCC950 and PD-MSC-CM (*n* = 2). It was observed that NLRP3 expression, which was monomeric in untreated OCI-AML2, was aggregated in multiprotein bodies as puncta upon LPS ATP stimulation (Supplementary Fig. S18). Further, PD-MSC-CM demonstrated a drastic reduction in NLRP3 intensity, indicating disaggregation of the assembly, comparable to that observed with MCC950 (Supplementary Fig. S18) 0.5*.*

#### In vivo efficacy of PD-MSC-CM-03

An exploratory study conducted to evaluate the growth reduction potential of PD-MSC-CM from (ETP-ALL-BM-MSC-CM) on leukemia xenografts in an immunodeficient mouse model demonstrated tumor reduction in PD-MSC-CM-treated mice compared to control mice (Supplementary Fig. S19-A). This trend was observed between days 15 and 24. It was interesting to note that the residual tumors in the efficacy experiment demonstrated that the growth-inhibitory effect was due to downregulation of NLRP3 pathway genes, viz. NLRP3, PYCARD, and IL-18, and to simultaneous upregulation of tumor suppressor genes, viz. CGREF-1, TP53, and PLK-5 (Supplementary Fig. S19-B). It was interesting to note that the BM-MSC (corresponding ETP-ALL-BM-MSC) treatment failed to exert effective tumor reduction (Supplementary Fig. S19-B). This data strongly corroborate the in vitro observations that MSC supports leukemia survival (Fig. 3D), whereas PD-MSC-CM inhibits leukemia growth (Fig. 5A–D).

**Fig. 6:**
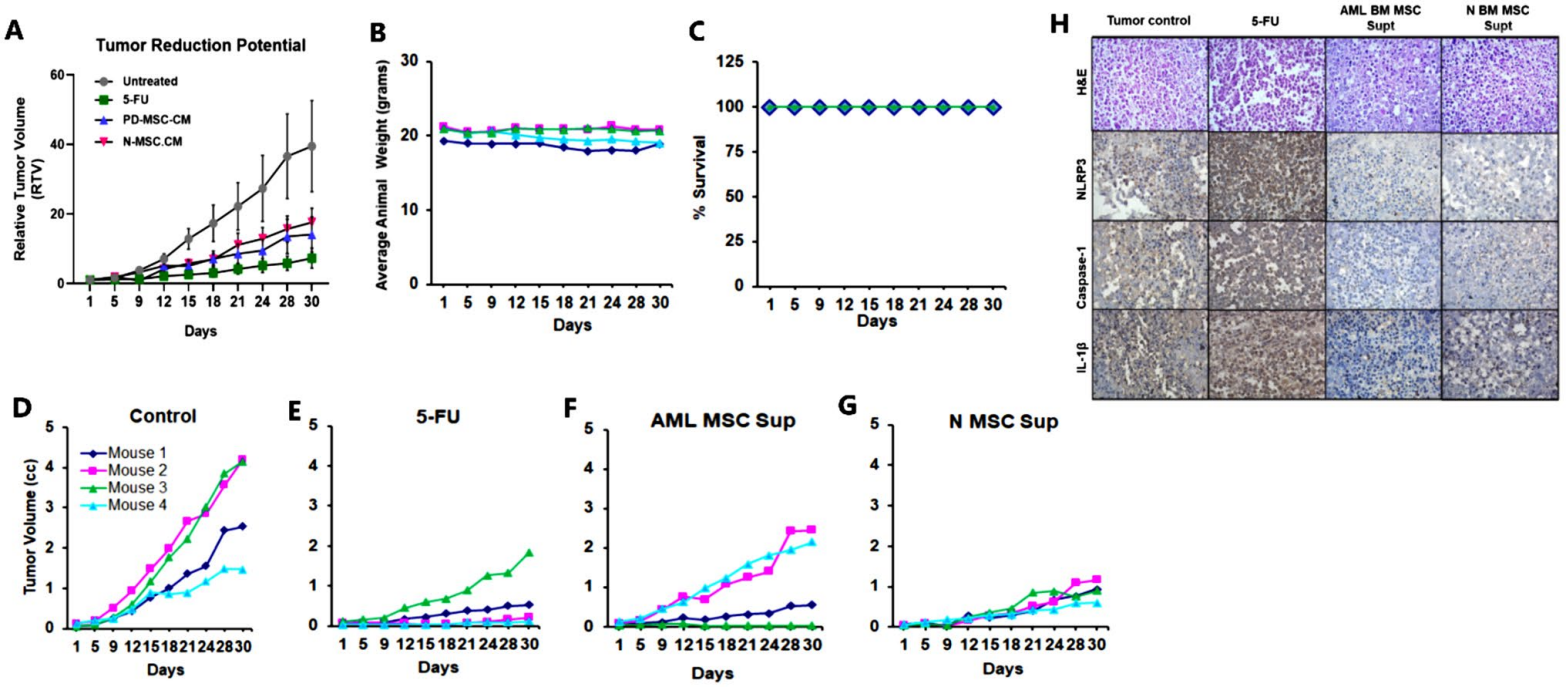
Effect of PD-MSC-CM on leukemia xenograft in immunodeficient mice: Panel **A** depicts a line graph representing relative tumor volumes of mice treated with MSC-CM. Panel represents **B** Average animal weight and **C** % survival, for the mice (*n* = 4) included in the respective groups during the study period. Panels **D**, **E**, **F**, and **G** show the tumor volume readings for individual mice within each group. Panel **H** represents the upright microscopic images of tumor sections stained for the expression of NLRP3 inflammasome markers isolated from NOD-SCID immunodeficient mice treated with PD-MSC-CM and N-MSC-CM. As per NCI guidelines, Toxicity Criteria: Mortality and weight loss ≥ 4 g/mouse indicate toxicity. In this study, mice in all three treatment groups did not show toxicity, indicating their safety in application, and 100% survival of experimental mice

Based on these leads, we conducted the main in vivo efficacy experiment using PD-MSC-CM-03 in NOD-SCID immunodeficient mice (*n* = 4 per group). It was observed that PD-MSC-CM-03 reduced K-562 leukemia xenograft growth, which was at par with the chemotherapeutic drug 5-FU (Table 1 and Fig. 6A and 6D–F). The growth inhibitory effect of PD-MSC-CM-03 started as early as day 1, while the growth inhibitory effect of 5-FU started from day 9. The N-MSC-CM also showed a reduction in growth. The body weight of mice (Fig. 6B) was unaffected, and survival (Fig. 6C) was 100% throughout the experimental duration, indicating that both PD-MSC-CM-03 and N-MSC-CM were safe for administration.

**Table 1:** Effect of PD-MSC-CM on K-562 leukemia xenograft tumor growth in immunodeficient NOD-SCID mice

Days	Tumor control	5-FU	PD-MSC-CM-03	N-MSC-CM
1	1	1	1	1
5	1	0.75	1.14	1.19
9	1	**0.33**	0.86	**0.23**
12	1	**0.30**	0.72	0.59
15	1	*0.20*	**0.39**	0.45
18	1	*0.17*	**0.41**	**0.40**
21	1	*0.19*	**0.39**	0.50
25	1	*0.19*	**0.35**	0.47
28	1	*0.16*	**0.37**	0.43
30	1	*0.18*	**0.36**	0.45

As per NCI USA guidelines (Geran et al. 1972) [27], Activity Criteria: T/C ≤ 0.2 (Italic) is considered to demonstrate highly significant activity. T/C ≤ 0.42 (Bold) is considered to demonstrate significant activity

NOD-SCID mice, *n* = 4 per group. Dosing protocol: Positive Control 5-Fluorouracil (5-FU): 5 mg/kg i.p. day 1–9; PD-BM-MSC-CM/N-BM-MSC-CM—i.p 200 µl every alternate day. RTV = Relative Tumor Volume = Tumor Volume on day of measurement/ Tumor Volume on day 1; T/C Ratio- Test/ Control RTV; i.p. = intraperitoneal

## Discussion

AML represents a blood malignancy comprising rapidly proliferating immature myeloid cells [7]. Leukemic cells are known to interact with soluble factors and cellular components within the microenvironment, playing a crucial role in leukemia cell survival, promoting leukemogenesis, albeit standard chemotherapy treatment [5, 8]. Despite advances in chemotherapy, 80–90% AML patients relapse after achieving complete remission. Hence, our study focused on identifying stromal alterations and evaluating the dynamic interconnections among leukemic blasts, mesenchymal stem cells, and their soluble factors.

AML-BM-MSC were reported to exhibit enhanced adipogenic differentiation and impaired osteogenic differentiation potential [29]. Corroborative data were reported by Geyh et al*.* [30] and also further demonstrated significant growth deficiency in AML-BM-MSC compared to healthy BM-MSC. In another study, AML-BM-MSC demonstrated differential cytogenetics and cytokine profiles compared to normal BM-MSC [13, 31, 32]. In line with these results, we observed that AML-BM-MSC exhibited reduced osteogenic differentiation and that its secretome showed elevated IL-6 and IL-8 levels. IL-6 secreted by AML-MSC was responsible for inducing epithelial-mesenchymal phenotype in AML cells via IL-6/JAK2/STAT3 pathway [9]. Further, we have shown that IL-6 confers a survival advantage to AML cells in co-cultures, acting as a protumorigenic factor.

Chronic inflammation is a hallmark of cancer, and NLRP3 is central to the inflammasome pathway, which is known to be associated with tumorigenesis and progression [33, 34]. In our study, we observed differential gene expression of NLRP3 inflammasome pathway markers—CASP-1, IL-1*β*, IL-18, PYCARD, and NLRP3—compared with Un-BM MSC. The NLRP3 expression in AML-BM-MSC was validated in our study. Corroboratively, this was also validated in clinical AML bone marrow samples (data not shown). Study by Zong et al. [35] demonstrated the role of the NLRP3 inflammasome pathway in AML progression via the IL-1*β* pathway. We report, for the first time, that NLRP3 pathway molecules are distinctly expressed in AML-BM-MSC.

Dysregulated gene expression profiles observed in AML-BM-MSC compared to Un-BM-MSC were mainly associated with Protein digestion and absorption, Focal adhesion, Proteoglycans in cancer, and NF-*κ*B signaling pathways, and were skewed toward inflammatory pathway genes. Heidi et al. [36] conducted RNA sequencing and reported similar observations with significant deregulation of adhesion molecules and proteoglycans. By GeneChip Whole Transcription (WT) Binato et al*.* [32] reported CCL2 and BMP4 as differentially expressed genes governing alterations in human MSC signaling. In contrast, Huang et al*.* [31] didn’t find any significant changes in gene expression between AML-BM-MSC and healthy BM-MSC.

In our study, we observed that BM-MSC provided a survival advantage to leukemia cells in co-culture assays, as evidenced by increased cell growth, elevated sIL-6 and sIL-8 secretion, and enhanced recruitment of leukemia cells toward MSC, leading to sustained mitochondrial transfer. This observation was corroborated by another study, which showed that MSC supported OCI-AML3 blast apoptosis resistance and survival upon cytarabine treatment. This pro-survival effect was attributed to oxidative phosphorylation inhibition and enhanced mitochondrial transfer [37]. Corroborative reports were also observed by Moschoi et al*.* [38] where the acquisition of functional mitochondria by AML cells from mouse- or human-derived stromal cells increased mitochondrial ATP production, reduced chemotherapy-induced mitochondrial depolarization, and conferred a survival advantage. This mitochondrial transfer could be attributed to upregulation of NOX2 via tunneling nanotubes mediated by superoxide Marlein et al*.* [39]. Another study reported MSC providing a survival advantage to leukemia blasts through immunosuppressor TGF-*β* secretion, which stabilized the leukemic niche by inducing p38 MAPK/ALDH2-mediated drug resistance [40]. Further, Brenner et al. [5] observed that the pro-tumorigenic effect of MSC was through the secretion of soluble factors that directly enhanced AML cell survival and therapy resistance. They performed co-culture studies demonstrating that MSC-derived cytokines, viz. IL-6, IL-8, and CXCL12 induced AML cell quiescence and activated the anti-apoptotic BCL-2 and Notch signaling pathways. Our results aligned with observations, that the protumorigenic effect of AML-BM-MSC on leukemia cell survival was mediated by increased sIL-6 and sIL-8.

In our study, PD-MSC-CM demonstrated an anti-leukemia effect and enhanced the chemosensitivity of leukemia cells to the drug cytarabine. To our knowledge, this is the first report and novel finding that drug resistance observed in the presence of AML-BM-MSC is successfully reversed in the presence of PD-MSC-CM. This effect of PD-MSC-CM observed in vitro was confirmed and validated in in vivo physiological mouse models. PD-MSC-CM was found to be immunomodulatory and enhanced NK-mediated killing of leukemia tumor targets. The in vivo efficacy of PD-MSC-CM in reducing leukemia xenograft was found to be through regulating the gene transcripts of CXCL8 and ADM (data not shown), which are known to be pro-tumorigenic [5]. Further, it was found to be safe in healthy immune cells and did not alter the mice’s body weight or affect their survival. These observations indicate that PD-MSC-CM is safe for administration and warrants further exploration in clinical trials. In one study reported by Silva et al. [41]. MSC-produced azurin in a CM configuration exerted an anticancer effect in vitro and in vivo breast cancer models. Here, the MSC-CM was derived from BM-MSC and UC-MSC, which were engineered and transfected with a plasmid encoding azurin, a cytotoxic granule. Another study demonstrated that MSC-CM derived from the BM of healthy individuals, along with exogenously added wortmannin, led to tumor-inhibitory potential in the breast cancer model [42]. Another study reported that adipose tissue-derived MSC from healthy individuals and their CM induced apoptosis in HeLa cervical cancer cells in vitro [43]. Their study emphasized that MSC-CM activated healthy PBL by increasing cytokine secretion (IL-2, IFN-*γ*, and TGF-*β*), thereby inducing cytotoxicity in HeLa cells through immunomodulatory effects. They concluded that the properties of MSC-CM collected from days 1–5 showed variable effects on PBL in co-culture with HeLa cells. In alignment with these observations, our study demonstrated that PD-MSC-CM increased PBL metabolic activity and enhanced immune cell-mediated killing of target leukemia cells. We observed that PD-MSC-CM were composed of key cytokines involved in the activation of *T*- and *B*-cells, viz. sIL-6, sIL-8, sIL-21 and sIL-23.

Freita et al*.* [44] observed growth inhibition of K-562 and K-562-lucena cell lines in vitro by co-culture of normal BM-MSC in a transwell co-culture system. They reported that the secretome of these MSC could be responsible for inhibiting leukemia cell growth. However, they failed to observe the same effect using AML-BM-MSC. They stated that, in their study, the mechanisms governing leukemia cell behavior remain to be investigated. Few studies have used UC-MSC-CM to demonstrate in vitro inhibition of hepatocellular carcinoma cell proliferation*.* Jin et al*.* [45] and breast cancer animal model *in vivo* Panahandeh et al. [14]. We also observed reduced IL-6 levels in PD-MSC-CM-treated OCI-AML2 cell supernatants, which may be attributed to IL-6’s protective effects in AML cells.

It was intriguing to note that PD-MSC-CM demonstrated significant tumor growth reduction in a leukemia xenograft mouse model, as per the criteria of the NCI, USA. The mice’s body weight and survival were not affected, indicating the safety of this composition in future experiments under the drug development pipeline. Zong et al*.* [35] demonstrated increased expression of NLRP3 in the AML murine model, which was significantly reduced in the NLRP3 knockdown model. Hence, in our study, the mechanistically anti-leukemia activity of PD-MSC-CM could be attributed to the regulation of NLRP3 pathway marker expression in the xenograft microenvironment. This was also confirmed and validated by immunohistochemistry and an in vitro inflammasome activation model.

In our parallel study, we have successfully reported a novel invention that, UC-MSC-CM exhibits immunomodulatory properties and reduced xenograft tumor growth in preclinical model of breast cancer, lung cancer and cervical cancer. This effect was found to be executed by reducing mice sIL-6 and sIL-1*β* secretions [46].

Major limitation of the study is the small sample size for each experiment. Since MSC are isolated from the BM of cancer patients, MSC themselves are not oncogenic and have a limited culture lifespan. Also not all BM cultures lead to primary cultures of MSC. Hence, the conditioned media per MSC per cancer patient is restricted, allowing only a few sets of experiments. Due to ethical restrictions, procuring normal BM is very challenging, and purchasing normal BM-MSC is very expensive. Consequently, N-BM-MSC-CM is available in limited quantities and can be used as a normal control in all experiments. Since this normal BM is mainly obtained from accidental injury cases, during the COVID-19 era, there was a significant supply shortage. Umbilical cord-derived MSC are comparatively readily available; however, they may not be functionally at par with adult bone marrow. Hence, in some experiments, we incorporated readily available uninvolved BM after obtaining ethical approval. Despite these hurdles, since we obtained novel leads, we have tried to confirm and validate our findings using multiple approaches, including immune-based, protein-based, genome-based, and preclinical in vivo experiments. In fact, our leads and findings demonstrate anticancer activity across leukemic models using conditioned media from bone marrow MSC sourced from healthy individuals (normal stroma), leukemia patients (dysregulated stroma), or lymphoma patients (uninvolved bone marrow).

## Conclusion

In summary, our study has provided interesting leads that leukemia patient-derived BM-MSC exhibit a pro-tumorigenic role. There is a paradigm shift in which PD-MSC-CM exhibit opposite roles, i.e., anti-leukemic and immunomodulatory activities. PD-MSC-CM composition showed distinct levels of secretory cytokines sIL-6, sIL-8, and sIL-21, and growth factors angiopoietin-1 and VEGF in all samples. In addition, PD-MSC-CM demonstrated cytocompatibility with healthy PBLs while enhancing NK cell-mediated killing of target K-562 cells, suggesting promising avenues for further exploration as immunomodulatory agents. Furthermore, we showed that PD-MSC-CM impaired leukemia cell growth by reducing mitochondrial membrane potential and altering the cell cycle, as observed in in vitro studies. This effect was further validated in vivo using a leukemia xenograft model in immunodeficient mice. To the best of our knowledge, this is the first comprehensive study to project the dual roles of AML-derived BM-MSC and PD-MSC-CM. For product development, scaling up, and treatment, pooling MSC-CM may be necessary, as the composition of all CM is similar; however, the quantities of immune cytokines or growth factors vary. Cytocompatibility and anti-tumor immunity function-enhancing ability of PD-MSC-CM warrants further development of this agent as an immunomodulatory anticancer agent for leukemia management. Our current study is innovative, and PD-MSC-CM, being a cell-free product, may pave the way for novel, “off-the-shelf” approaches in treating Leukemia.

## Supplementary Information

Below is the link to the electronic supplementary material.

Supplementary file1 (DOCX 41 KB)

Supplementary file2 (DOCX 12093 KB)

Supplementary file3 (DOCX 40 KB)

Supplementary file4 (MP4 11770 KB)

Supplementary file5 (MP4 8160 KB)

## Data Availability

Data will be made available upon request. The microarray sequences have been submitted and registered in the NCBI-GEO database Accession Series # GSE150070 (GSM4522998 to GSM4523013), 8th May 2020.

## Abbreviations

5-FU: 5-Florouracil
A2AR: Adenosine A2A receptor
A2BR: Adenosine A2B receptor
ADR: Adriamycin
AML: Acute myeloid leukemia
AML-BM-MSC: Acute myeloid leukemia mesenchymal stem cells
ATP: Adenosine tri-phosphate
BM: Bone marrow
CBA: Cytokine bead array
CCCP: Carbonyl cyanide m-chlorophenylhydrazone
CD: Cluster of differentiation
CM: Conditioned media
DGE: Differential gene expression
EGF: Epidermal growth factor
ER: Endoplasmic reticulum
ETP-ALL: Early T-cell precursor acute lymphocytic leukemia
FDR: False discovery rate
FGF: Fibroblast growth factor
GSEA: Gene set enrichment analysis
IFN-*β*: Interferon beta
IFN-*γ*: Interferon gamma
IL-1*β*: Interleukin-1 beta
LPS: Lipopolysaccharide
MCP-1: Monocyte chemoattractant protein-1
MSC: Mesenchymal stem cells
MTT: 3-(4,5-dimethylthiazol-2-yl)-2,5-diphenyltetrazolium bromide
N-BM-MSC: Normal bone marrow mesenchymal stem cells
NES: Normalized enrichment score
NHL: Non-Hodgkin lymphoma
NLRP3: NOD like receptor family pyrin domain containing 3
OCI-AML2: Ontario Cancer Institute—acute myeloid leukemia 2
ORA: Over representation analysis
PD-MSC-CM: Patient-derived mesenchymal stem cell conditioned media from AML patients
PECAM-1: Platelet endothelial cell adhesion molecule-1
PlGF: Placental growth factor
*q*-PCR: Quantitative real time polymerase chain reaction
RTV: Relative tumor volume
sIL-6: Soluble interleukin-6
T/C ratio: Test/control ratio
TEM: Transmission electron microscopy
TIM-3: T-cell immunoglubulin and mucin-domain containing-3
TNF-*α*: Tumor necrosis factor alpha
UC-MSC: Umbilical-cord Wharton jelly derived mesenchymal stem cells
Un-BM: Uninvolved bone marrow
VEGF: Vascular endothelial growth factor
