# Supplementary material for: Defining alteration in bone marrow mesenchymal stem cells (MSC) from acute myeloid leukemia and exploring cultured MSC-conditioned media as a novel anti-leukemia therapy agent
Source: Cancer Immunol Immunother. 2026 Feb 23;75(3):83. doi: 10.1007/s00262-025-04262-2 (PMC12929743; doi:10.1007/s00262-025-04262-2)
Supplement: Supplementary file 1 — Supplementary file1 (DOCX 41 KB) [file 262_2025_4262_MOESM1_ESM.docx]

**Defining alteration in bone marrow mesenchymal stem cells (MSC) from acute myeloid leukemia and exploring cultured MSC-conditioned media as a novel anti-leukemia therapy agent**

Manasi Nagare**^1,10^,** Monalisa Sahoo**^1^**, Manju Sengar**^3,10^**, Sachin Punatar**^3,10^**, Navin Khattry**^3,10^**, Anant Gokarn**^3,10^**, Bhausaheb Bagal**^3,10^**, Hasmukh Jain**^3,10^**, Sumeet Mirgh**^3,10^,** Sridhar Epari**^4,10^**, Tanuja Shet**^9,10^**, Trupti Pradhan**^1^**, Shweta Shirsat**^1^**, Madan Barkume**^2^**, Caroline Mathen^11^, Poonam Gera**^7,10^**, Rohit Kumar Verma**^6^**, Elveera Saldanha**^6,10^**, Pratik Chandrani**^6,10^**, Jitendra Gawde**^8^**, Shubhada Chiplunkar**^5,10^**, Jyoti Kode**^1,10*^**

- 1. **Cell lines and reagents used in the study**

OCI-AML2 cells were maintained in complete MEM-α supplemented with 20% FBS, 2.5mM HEPES, and 1% antibody cocktail (PSGM). HL-60, KG-1, and K-562 cell lines were procured from the National Cancer Institute, USA, and maintained in RPMI 1640 supplemented with 10% FBS, 1% antibiotics, and 1% L-glutamine. K-562 cells were used to generate a xenograft in NOD-SCID mice to assess the tumor-inhibitory potential of PD-MSC-CM. Umbilical cord Wharton jelly-derived-MSC (UC-MSC) were procured from OCT Therapies and Research Pvt Ltd, Mumbai) included as a control for mechanistic studies. UC-MSC were maintained in complete DMEM with 10% growth supplement and 0.5% antibiotics. Normal Bone marrow mesenchymal stem cells (N-BM-MSC) were commercially procured (Hi-Media Laboratories Pvt Ltd, India), as a comparison control for PD-MSC-CM experiments and maintained in DMEM supplemented with 1% antibiotic cocktail and 10% FBS. Details of the reagents are provided in Supplementary Table ST1.

Immunophenotypic characterization of cultured AML-BM-MSC was conducted by staining with fluorochrome-conjugated anti-human antibodies against different markers (Supplementary table ST1): CD90, CD73, CD105, TIM-3, and CD45. The percent viability of OCI-AML2 cells was determined using the MTT assay. The cell-growth-inhibitory effect of PD-MSC-CM was assessed by Sulforhodamine B staining. Cell cycle analysis was performed by fixing cells in 70% ethanol, treating them with RNase A, and staining with propidium iodide. Mitochondrial membrane potential in OCI-AML2 cells was evaluated by JC-1 staining. Cytarabine was used to study the role of AML-BM-MSC in OCI-AML2 cell fate via direct cell-cell and cell-free interactions.

- 1. **Sample processing, enrichment, and maintenance of BM-MSC**

BM sample (3-5ml) was processed by incubating in the Rosette-Sep human mesenchymal stem cell enrichment cocktail (Stem cell technologies, Canada) for 30 minutes in the dark at room temperature. Further, samples were diluted with normal saline (0.85% NaCl) and layered onto a Ficoll-Hypaque gradient. Samples were centrifuged at 1500 rpm for 20 minutes at room temperature. Buffy coat was collected in fresh culture tubes, washed with normal saline, and centrifuged (Eppendorf 5801 R, Eppendorf, Germany) at 1000 rpm for 10 minutes. Cell viability was estimated by the trypan blue dye exclusion method. Isolated cells were seeded in a T75 flask at a density of 40x10^6^ cells per flask and allowed to adhere for 24 hours. The following day, the non-adherent cells were removed, and adherent populations were maintained in Minimum Essential Medium-alpha supplemented with human mesenchymal stem cell stimulatory supplement (Stem cell technologies, Canada). MSC could be maintained up to passages P3/P5 and used for experiments.

**2.5. Characterization of AML-BM-MSC**

**2.5.1. Immunophenotypic identification of patient-derived AML BM-MSC**

AML-BM-MSC cultured up to passage P3/P5 were trypsinized, washed, and fixed with 1% PFA for 15 minutes at 4°C. Fixed cells were suspended in 50 µl FACS buffer (1xPBS + 1% FBS + 0.1% Sodium Azide) and incubated with fluorochrome-conjugated anti-human antibodies against CD45, CD90, CD73, and CD105 for 45 minutes at 4°C. Expression of additional markers, viz. Immune checkpoint receptor TIM-3, Toll-like receptors (TLR-3, TLR-4), and adenosine receptors (A2AR and A2BR) were also evaluated. Cells were resuspended, washed with FACS buffer, and centrifuged at 1000 rpm for 10 minutes. Stained cells were acquired by a flow cytometer (FACS Aria III, BD Biosciences, USA). Data were analyzed using FlowJo v10 software (BD Biosciences, USA).

- - 1. **Morphological characterization of cultured MSC**

Cultured AML-BM-MSC at passage P2/P5 and UC-MSC were studied for their morphological characteristics under a bright field microscope and for ultrastructural alterations by TEM. MSC were seeded at a density of 5x10^4^ cells in a 35 mm petri-dish and incubated at 37°C to obtain 70-80% confluency. Similarly, to evaluate the effect of OCI-AML2 cells on UC-MSC ultrastructural morphology, MSC were cultured as single cells and in co-cultures with OCI-AML2 for 24 hours. For ultrastructural analysis, the monolayer cultured cells were fixed with 3% glutaraldehyde for 2 hours at 4°C and post-fixed in 1% aqueous osmium tetroxide for 1 hour at 4°C. Fixation was followed by en-block staining in 2% aqueous uranyl acetate. The cells were then subsequently dehydrated in increasing grades of alcohol for 10 minutes each. The cells were further infiltrated with an increasing series of HPMA, followed by Epon resin infiltration for 15 minutes each, and embedded in Epon resin for polymerization at 60°C for 48 hours. The cells from the polymerized Epon sheet were marked, and the sheet was cut into small pieces, which were re-embedded on the pre-polymerized block and allowed to polymerize. The ultrathin sections of 70nm were cut using a Leica UC7 ultramicrotome and collected on 150 mesh copper grids. The sections were contrasted with lead citrate, and images were acquired using JEM 1400 Plus Transmission Electron Microscope (JEOL, Japan) at 120kV and analyzed using i-TEM Analysis Software (Olympus). Quantification of mitochondria and intracellular vesicles in UC-MSC vs AML-BM-MSC, and demonstrating alteration of healthy UC-MSC after co-culture with OCI-AML2, was performed by enumerating the individual number of mitochondria and intracellular vesicles in cells after co-culture by i-TEM Analysis Software from Olympus. Images were acquired at 10000x magnification, and the number of mitochondria and vesicles per section (n=5) was counted manually.

- - 1. **Multi-lineage differentiation potential of AML-BM-MSC**

AML-BM-MSC cultured up to passage P3 were evaluated for their multilineage differentiation potential into osteogenic and adipogenic lineages. AML-BM-MSC were cultured in Mesencult basal medium supplemented with osteogenic and adipogenic stimulatory supplements and incubated at 37 °C for 21 days. The medium was changed after every other alternate day. Cells were washed with 1xPBS and fixed with ethanol for 30 minutes at 4°C. Differentiated AML-BM-MSC were stained with Oil red O stain for adipogenic differentiation and alizarin red S for osteogenic differentiation.

- - 1. **Gene expression profiling in AML-BM-MSC**

AML-BM-MSC (n=2) were evaluated for their differential gene expression in comparison to Un-BM-MSC (n=2). Briefly, Un-BM-MSC and AML-BM-MSC cultured up to passages P2 to P3 were fixed in RNA later and submitted to Genotypic Technologies Pvt Ltd. to study the differential gene expression profiling. RNA was extracted using Qiagen’s RNeasy mini-kit as per the manufacturer’s protocol. The samples for Gene expression were labeled using the Agilent Quick-Amp labeling Kit (p/n5190-0442). The total RNA was reverse transcribed at 40°C using oligo dT primer tagged to a T7 polymerase promoter and converted to double-stranded cDNA. Synthesized double-stranded cDNA was used as a template for cRNA generation by *in vitro* transcription and the dye Cy3 CTP (Agilent) was incorporated during this step. The cDNA synthesis and *in vitro* transcription steps were carried out at 40°C. Labeled cRNA was cleaned up using Qiagen RNeasy columns (Qiagen, Cat No: 74106) and quality assessed for yields and specific activity using the Nanodrop ND-1000. Fragmentation of labeled cRNA and hybridization were done using the Gene Expression Hybridization kit of Agilent Technologies (In situ Hybridization kit, Part Number 5190-0404). Hybridization was carried out in Agilent’s Surehyb Chambers at 65ºC for 16 hours. The hybridized slides were washed using Agilent Gene Expression wash buffers (Agilent Technologies, Part Number 5188-5327), scanned using Agilent Microarray Scanner (Agilent Technologies, Part Number G2600D). Data extraction from Images was performed using Feature Extraction software, and data normalization was performed in GeneSpring GX using the 75th percentile shift method, and gene-based analysis was performed. The sequences have been submitted and registered in the NCBI-GEO database Accession Series # GSE150070 (GSM4522998 to GSM4523013), 8^th^ May 2020.

**Analysis:** Gene Set Enrichment Analysis (GSEA) was performed using the WebGestalt online tool (<https://www.webgestalt.org/>). The gene names and the rank list were uploaded. Finally, the KEGG pathway database was selected. The parameter was considered as pathways with FDR $\leq$0.05, *p*-value < 0.05, and absolute normalized enrichment score (|NES|) > 1 were considered as statistically significant. The analysis was set to 10,000 permutations. WebGestalt identified enriched KEGG pathways with associated enrichment scores, normalized enrichment scores (NES), and FDR values.

- 1. **Validation of expression of inflammasome pathway genes in AML-BM-MSC by quantitative real-time polymerase chain reaction**

Validation of the expression of inflammasome markers in AML-BM-MSC was conducted using quantitative real-time polymerase chain reaction. RNA was extracted from AML-BM-MSC and UC-MSC using the phenol-chloroform method, followed by precipitation of RNA using isopropyl alcohol. Briefly, MSC were lysed in guanidium thiocyanate-phenol solution. Further, chloroform was added at a TRIzol: chloroform ratio of 5:1. The mixture was centrifuged for 15 mins at 14000 rpm at 4˚C to facilitate phase separation. The upper aqueous phase was collected in a fresh Eppendorf tube, and an equal volume of isopropyl alcohol was added to precipitate RNA. Samples were stored at -80˚C overnight, then centrifuged at 14000 rpm for 10 minutes to obtain an RNA pellet. This pellet was washed in increasing ethanol gradients (80% and 100% ethanol) and centrifuged at 14000 rpm for 10 minutes each. Pellet was air dried and resuspended in RNase-free water (DEPC-treated water) and used to synthesize cDNA using Revert Aid First Strand cDNA Synthesis Kit (Thermo Scientific, Country) as per the manufacturer’s protocol. Quantitative expression of inflammasome markers, viz. NLRP3, IL-18, and Caspase-1 were performed using Kapa SYBR® FAST qPCR Master Mix (2X) kit as per the manufacturer’s protocol. 18S rRNA was used as a housekeeping gene for normalization. Experiments were conducted in three technical triplicates for two biological samples. Gene expression was calculated using the relative quantification method with the 2-ΔΔCT method. Primer sequences are provided in the Supplementary Table ST3.

- 1. **Characterization of MSC-CM derived from AML patients**

*Multiplex Luminex assay*

Media composition of PD-MSC-CM from the BM of healthy and AML patients was evaluated by AimPlex multiplex assay. CM was added to a 96-well filter plate (available in the kit) containing premix beads and incubated on a plate shaker (700rpm) at R.T. for 60 minutes. After incubation, plate was washed 3x with wash buffer and further incubated with biotinylated antibody (incubation on shaker for 30 minutes at R.T) and Streptavidin-PE (incubation on shaker for 20 minutes at R.T.) with subsequent washes. Samples were re-suspended in 1X Reading buffer and acquired using flow cytometer (Beckman Coulter, DX FLEX autoloader, USA). Data was analyzed using Soft Flow’s FCAP Array v3 or Infinite.

- 1. **Evaluation of inflammatory cytokine profile in AML-BM-MSC-CM and co-culture supernatants**

*Cytokine bead array*

To study the role of AML-BM-MSC in AML cell response in cell-cell direct and indirect cultures, OCI-AML2 were cultured in single or in the presence of AML-BM-MSC for 48 hours. Resulting culture supernatants were collected, centrifuged at 1000 rpm for 10 minutes, and supernatants were stored at -80°C and used for identification of inflammatory cytokine profile by Human Inflammatory Cytokine CBA Kit (BD Biosciences, USA). Briefly, capture beads for individual cytokines were mixed in a 15 ml Falcon to obtain a mixture of beads. Capture beads (2.5µl) mix was then distributed in assay tubes. Further equal volumes of samples and Human Inflammatory Cytokine PE Detection Reagent (2.5µl) were added to assay tubes, and samples were incubated for 3 hours at room temperature, protected from light. After incubating, samples were washed with 1 ml wash buffer and centrifuged for 10 minutes at 1000 rpm. Supernatant was carefully discarded, and the pellet was resuspended in wash buffer. Samples were acquired by flow cytometer (FACS ARIA I). Acquired samples were analyzed by FCAP Array Administrator, BD FACS Aria (BD Biosciences, USA).

- 1. **Effect of AML-BM-MSC and PD-MSC-CM on AML cell growth potential by *in vitro* assays**

To assess the effect of AML-BM-MSC on OCI-AML2 cells, OCI-AML2 cells were cultured in single and co-cultures at an AML-BM-MSC: OCI-AML2 ratio of 1:10 in the presence of cytarabine for 48 hours. As a positive control, Adriamycin (broad-spectrum anti-cancer agent) – ADR at a concentration of 1µM was used for comparison. After incubation, OCI-AML2 cells were collected, counted for viability using the trypan blue method, and seeded in a 96-well plate and incubated with MTT reagent for 4 hours at 37°C. Cell viability was calculated by measuring the absorbance at 540nm.

To assess the cytotoxic effect of PD-MSC-CM, OCI-AML2 cells were cultured in the presence of CM for 48 hours. The cytotoxic potential of cytarabine against CM-treated OCI-AML2 was also included. Briefly, OCI-AML2 cells were seeded at a density of 5000 per well in 96 flat-bottom microtiter plates and cultured in PD-MSC-CM containing cytarabine (1 and 2 µM), incubated at 37°C for 48 hours under humidified CO_2_ conditions. Cells cultured in the presence of regular MEM-α medium served as controls. At the end of the incubation, cells were fixed with 50% TCA and incubated at 4°C for 1 hour. Precipitated cellular proteins were stained with SRB dye and incubated at room temperature for 20 minutes on a rocker. Plates were washed with acetic acid to remove unbound dye, and bound dye was eluted using 10mM Tris base. Absorbance was recorded at 565nm, and percent growth was calculated.

- 1. **Co-culture interactions between MSC and AML cells by real-time confocal microscopic imaging**
     1. *Transwell Assay*

Recruitment of OCI-AML2 cells to AML-BM-MSC was determined by trans well assay. Briefly, AML-BM-MSC were seeded in the lower chamber of a 24-well plate at a density of 5x10^3^ per well, and cells were allowed to adhere overnight. The following day, OCI-AML2 cells suspended in plain media were seeded in trans well inserts and placed in the wells containing AML-BM-MSC (in 2% FBS). Trans well inserts containing OCI-AML2 cells with Cytarabine 1 and 2 µM were also included to understand the influence of drug treatment on the migratory potential of leukemia cells. Co-cultures were incubated for 24 hours at 37˚C under humidified CO_2_ conditions. After incubation, trans well inserts were separated, and the migrated cells in the lower chambers were enumerated. Cells were counted for 7 consecutive sections in every treated well, and the data were analyzed using ImageJ software (Company, USA).

- - 1. *Live cell monitoring by laser confocal microscopy*

Study of AML cell recruitment to AML-BM-MSC: To study the interactions between co-cultures, AML-BM-MSC were labelled with Mito tracker CMX ROS red, and OCI-AML2 cells with PKH67 green. Co-cultures were set up with an MSC: AML ratio of 1:10, and interactions were monitored by live cell imaging using 3i spinning disc confocal microscope. Cell-cell interaction data was analyzed using Imaris software.

- - 1. *Mitochondrial transfer ability of AML-BM-MSC*

To study the mechanism of mitochondrial transfer from MSC to AML cells, MSC and OCI-AML2 cells were stained with Mitotracker CMX-ROS and PKH67 green. Stained cells were co-cultured for 1 to 4 hours, and live cell imaging was performed using the Incucyte S3 live cell analysis system. To demonstrate mitochondrial transfer between MSC and OCI-AML2 cells, co-cultures were initiated using Mitotracker CMX ROS-stained MSC (AML-BM-MSC or UC-MSC) and PKH67 green-stained OCI-AML2 in the presence/absence of cytarabine. Live cell imaging was conducted using 3i spinning disc Confocal microscope at a magnification of 100x. Data was analyzed using Incucyte software.

- 1. **Evaluation of the effect of AML-BM-MSC and PD-MSC-CM on AML cell mitochondrial membrane potential in AML cells**

To evaluate the effect of AML-BM-MSC or PD-MSC-CM on mitochondrial integrity in OCI-AML2, cells were cultured in the presence of cytarabine either as a single/co-culture/in the presence of PD-MSC-CM for 24 hours. After incubation, OCI-AML2 cells were collected from single and co-cultures, washed with 1x by centrifuging at 1000 rpm for 10 minutes. Cells treated with Carbonyl Cyanide m-Chlorophenyl Hydrazone (CCCP) at a concentration of 5µM were used as a positive control to represent a reduction in mitochondrial membrane potential. Washed cells were stained with lukewarm staining solution composed of 1xPBS and 5,5′,6,6′-Tetrachloro-1,1′,3,3′-tetraethylbenzimidazolylcarbocyanine iodide (JC-1) at a concentration of 1 µM and incubated at 37°C for 10-15 minutes. Stained cells were washed with 1x PBS to obtain a stained pellet, which was suspended in 1x PBS and acquired using Attune NxT (ThermoFischer Scientific, USA).

- 1. **Evaluation of the effect of PD-MSC-CM on inflammasome protein expression in AML cells**

The effect of PD-MSC-CM on NLRP3 protein expression was evaluated by the indirect immunofluorescence method. OCI-AML2 cells were stimulated with LPS (1µg/ml) for 4 hours and ATP (5mM) for 45 minutes to induce inflammasome activation. Cells treated with 5µM for 1 hour before ATP treatment were used as a positive control for NLRP3 inhibition. Treated cells were fixed with chilled methanol for 5 minutes at 20˚C. Fixed cells were permeabilized with 0.1% Triton-X 100 buffer (0.1% Triton-X-100 in 1xPBS) for 30 minutes at r.t followed by blocking (3% BSA in 1xPBS). Cells were incubated with an antibody specific to NLRP3 (Company, location) overnight at 4˚C. The following day, cells were washed and incubated with secondary antibody anti-rabbit AF488 (Company, location) for 1 hour at r.t. OCI-AML2 cells were counterstained with DAPI for 20 minutes. Cells were mounted using Vectashield mountant (Dako, Denmark), and data were acquired using an LSM-980 laser confocal microscope (Carl Zeiss AG, Germany). Data was analyzed using Fiji ImageJ (USA).

- 1. **Evaluation of the effect of AML-BM-MSC and PD-MSC-CM on AML cell cycle phases in AML cells**

OCI-AML2 cells were cultured in single/co-cultured/in the presence of PD-MSC-CM with cytarabine (1 µM) and incubated at 37°C for 24 hours. After incubation completion, OCI-AML2 cells were collected and washed with 1xPBS, and pellets were disrupted to obtain homogenously distributed cells. 70% Ethanol (Chilled) was added dropwise to cells with simultaneous vortexing to ensure uniform fixation. For cell cycle analysis, fixed cells were washed twice with 1xPBS to remove any residual ethanol and incubated with 4µg/ml propidium iodide and 10 µg/ml of RNase A solution for 15 minutes at 37°C. Cells were immediately acquired using Attune-NxT (Thermo Fischer Scientific, USA).

- 1. **To study the effect of PD-MSC-CM on leukemia tumor growth in immunodeficient mice model**

The cryopreserved KG-1/K-562 xenograft tumors were passaged to obtain donor mice. For the induction of tumor, small pieces of ~2-3 mm xenografts were subcutaneously implanted onto the flank region of NOD-SCID mice. When the average tumor volume reached approximately. 50 to 60 mm³, the mice were randomized into desired groups (n=4/group) before start of the treatment. viz. control (A), positive 5-FU treated group (B), N-BM-MSC-CM treated (C) and PD-MSC-CM 03 treated (D) group. Mice in the positive group were treated with 5mg/kg 5-FU up to 9^th^ day via the intraperitoneal route. Mice in groups (C) and (D) were administered 200µl/mouse CM thrice a week. Mice were monitored every fourth day for body weight, tumor volume, and mortality. Tumor volume was measured using the Vernier calliper. Mice were sacrificed on the 31^st^ day, and tumors were collected and fixed for further analysis. The data was represented as Relative Tumor Volume (RTV in cc), T/C (ratio of test versus control) and survival. Tumor volume was calculated using the formula [(w1 × w1 × w2) × (π/6)], where w1 and w2 are the smallest and the largest tumor diameters (cm), respectively. RTV was measured as tumor volume on the day of measurement/ tumor volume on day 1. The T/C ratio is an indication of antitumor effectiveness. The percentage treatment/control (T/C%) values or percent tumor regression values were calculated as follows:

Relative Tumor Volume (RTV)

T/C= RTV STest/ RTV Control

Tumor Regression %= 100- [ T/C*100]

T = mean tumor volume of the drug-treated group; RTV = mean tumor volume of the drug-treated group on the study day of interest – mean tumor volume of the drug-treated group on the initial day of dosing; C = mean tumor volume of the control group. As per NCI, USA guidelines, biological activity was considered significant when T/C values were ≤ 0.42 and highly significant when T/C values were ≤0.2. The percentage of survival was calculated at the end of the study for each animal and each compound administered. On the day of sacrifice, tumor pieces were preserved in formalin for histopathology.

- 1. **Validation of immune markers by Immunohistochemistry**

Tumor tissues from xenografts were fixed 10% neutral buffer formalin containing fixation solution. The tissues were processed and embedded in paraffin using conventional systems. The blocks were to obtain serial 4 μm thick sections and stained with conventional hematoxylin-eosin (H &amp; E) and also for markers specific to the inflammasome pathway markers NLRP3, IL-1β, and CASP-1. To evaluate the protein expression of inflammasome pathway proteins in leukemia xenograft, isolated tumor formalin-fixed paraffin-embedded blocks were processed to obtain sections, which were processed further for immunohistochemical analysis. Briefly, FFPE sections were de-paraffinized by heating at 60°C for 20 minutes, followed by treatment with Xylene for 10 minutes x3 at room temperature. Following deparaffinization, cells were rehydrated by treating with 50% ethanol X3 for 10 minutes at room temperature. Further, slides were washed under running water for 20 minutes and incubated in 1X Antigen Retrieval Buffer (ARB) for 20 minutes at 98°C and allowed to cool at room temperature. Peroxidase blocking was performed by treating cells with Peroxidase blocking buffer (Envision FLEX Mini Kit, Dako, Denmark) for 20 minutes at room temperature in a moist chamber. Slides were washed with wash buffer for 10 minutes on a rocker and primary antibody against NLRP3, IL-1β, and Caspase-1. Slides were incubated at room temperature for 1 hour and overnight at 4°C in a moist chamber. The following day, the Slides were washed and incubated with HRP-conjugated secondary antibody for 30 minutes at room temperature. Following washes x2 for 5 minutes each on rocker, slides were incubated with DAB chromogen at room temperature for 10 minutes at room temperature in dark conditions. Slides were washed and stained with counterstain hematoxylin for 5 minutes at room temperature. Slides were washed alternatively in the sequence Milli-Q, Wash buffer, Milli-Q, 5 minutes each and dehydrated by treating in increasing Ethanol gradients (50%, 70%, Absolute ethanol) for 5 minutes each. Further, slides were dehydrated by treating with a 1:1 ratio of xylene: alcohol solution and subsequent incubation in xylene x3 for 5 minutes each. Sections were mounted in DPX mountant and incubated at 37°C for 1 hour. Changes in tissue architecture in control versus treated tumors were recorded under a bright light microscope (Carl Zeiss Microscope, Zeiss Inc., Germany).

- 1. **Immunoassay to estimate the expression of secretory IL-18 levels in AML plasma samples at diagnosis**

ELISA kits were used to quantify expression of secretory plasma IL-18 levels as per the manufacturer’s protocol. Absorbance was measured at 450nm, and cytokine concentration (pg/ml) was evaluated.
