# Supplementary material for: Defining alteration in bone marrow mesenchymal stem cells (MSC) from acute myeloid leukemia and exploring cultured MSC-conditioned media as a novel anti-leukemia therapy agent
Source: Cancer Immunol Immunother. 2026 Feb 23;75(3):83. doi: 10.1007/s00262-025-04262-2 (PMC12929743; doi:10.1007/s00262-025-04262-2)
Supplement: Supplementary file 2 — Supplementary file2 (DOCX 12093 KB) [file 262_2025_4262_MOESM2_ESM.docx]

**Defining alteration in bone marrow mesenchymal stem cells (MSC) from acute myeloid leukemia and exploring cultured MSC-conditioned media as a novel anti-leukemia therapy agent**

Manasi Nagare**^1,10^,** Monalisa Sahoo**^1^**, Manju Sengar**^3,10^**, Sachin Punatar**^3,10^**, Navin Khattry**^3,10^**, Anant Gokarn**^3,10^**, Bhausaheb Bagal**^3,10^**, Hasmukh Jain**^3,10^**, Sumeet Mirgh**^3,10^,** Sridhar Epari**^4,10^**, Tanuja Shet**^9,10^**, Trupti Pradhan**^1^**, Shweta Shirsat**^1^**, Madan Barkume**^2^**, Caroline Mathen^11^, Poonam Gera**^7,10^**, Rohit Kumar Verma^6^, Elveera Saldanha^6,10^, Pratik Chandrani^6,10^, Jitendra Gawde^8^, Shubhada Chiplunkar^5,10^, Jyoti Kode**^1,10*^**

| **Figure** | **Page No.** |
| --- | --- |
| Supplementary Figure S1 | 2 |
| Supplementary Figure S2 | 3 |
| Supplementary Figure S3 | 4 |
| Supplementary Figure S4 | 5 |
| Supplementary Figure S5 | 6 |
| Supplementary Figure S6 | 7 |
| Supplementary Figure S7 | 7 |
| Supplementary Figure S8 | 8 |
| Supplementary Figure S9 | 8 |
| Supplementary Figure S10 | 9 |
| Supplementary Figure S11 | 10 |
| Supplementary Figure S12 | 10 |
| Supplementary Figure S13 | 11 |
| Supplementary Figure S14 | 11 |
| Supplementary Figure S15 | 12 |
| Supplementary Figure S16 | 13 |
| Supplementary Figure S17 | 14 |
| Supplementary Figure S18 | 15 |
| Supplementary Figure S19 | 15 |
| Supplementary Video SV1 | 16 |

**Supplementary Figure S1**


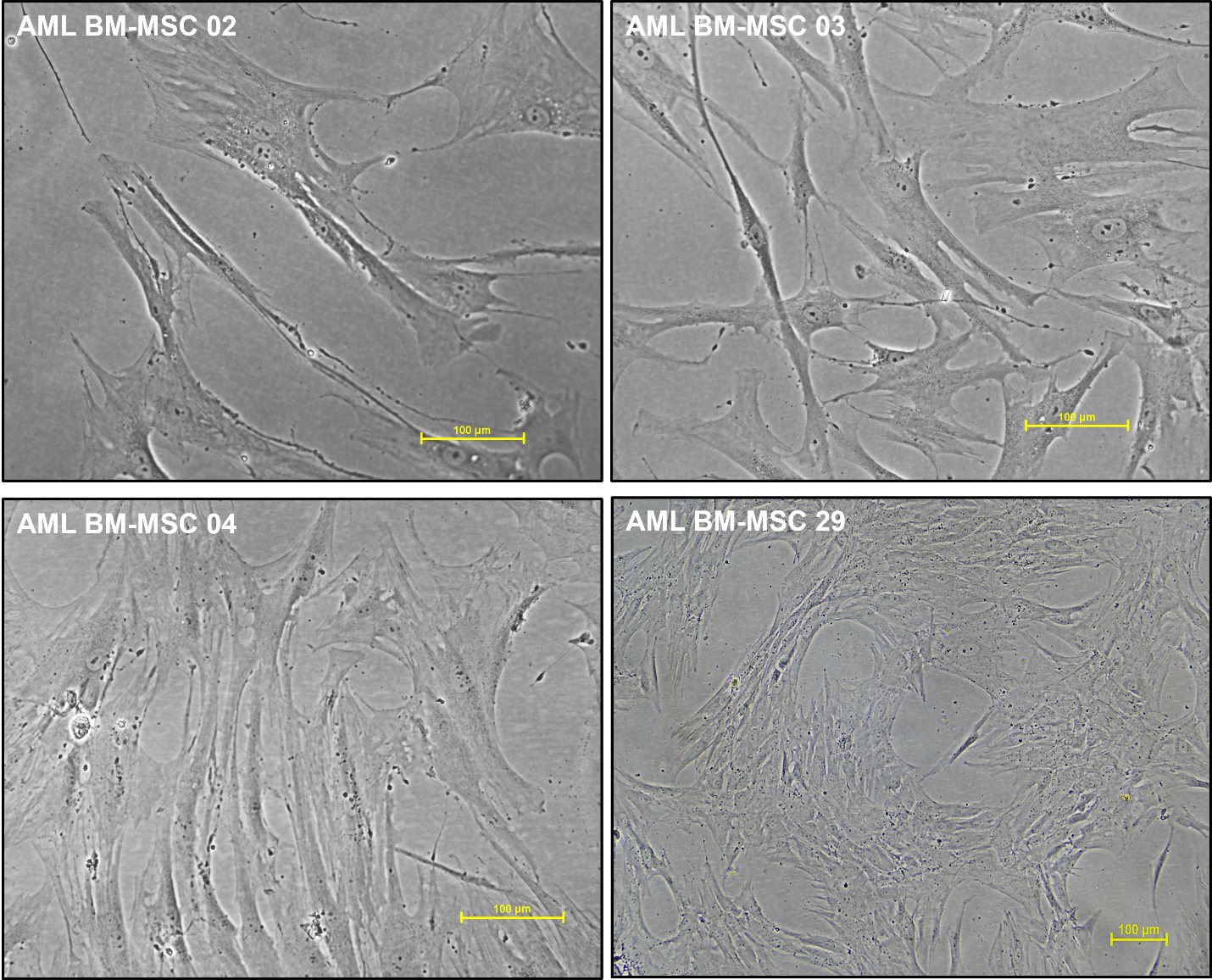


**Fig. S1 Morphological evaluation in AML-BM-MSC.** The figure depicts the bright-field microscopic images of mesenchymal stem cells isolated from AML bone marrow. AML-BM-MSC isolated from 4 different patients were cultured up to passage P5, and images were captured at a scale of 100µm with 20x magnification.

**Supplementary Figure 2**:


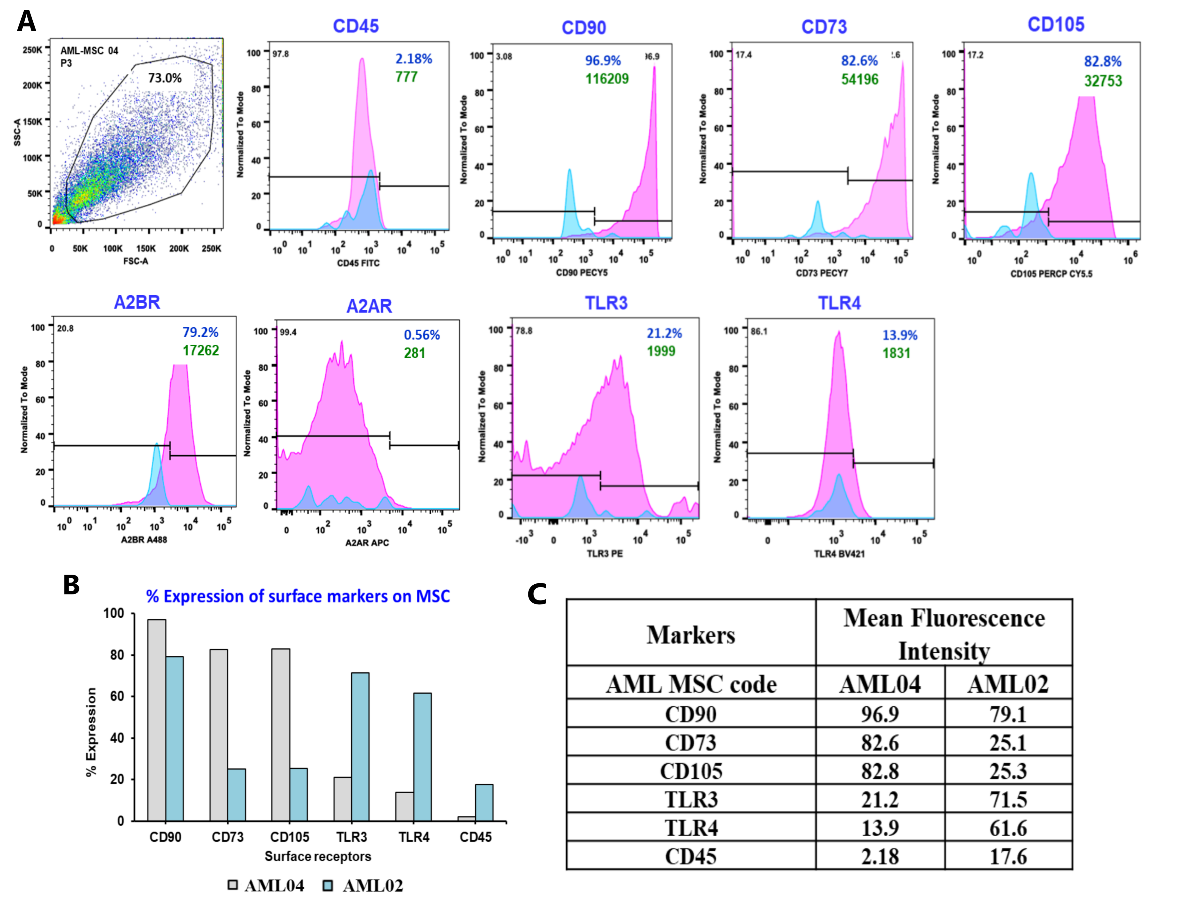


**Fig. S2 Multicolor immunophenotyping of AML-BM-MSC** Panel **[A]** depicts a scatter plot of isolated and cultured AML-BM-MSC and histogram plots describing mean fluorescence intensities (MFI) of expressed markers. Top right labels in blue indicate percent positive cells, and green labels represent MFI values. Panel **[B]** and **[C]** represent bar graph marker expressions in AML patient-derived BM-MSC and their representative MFI values in the table. The experiment was conducted in (n=3) biological samples.

**Supplementary Figure S3**


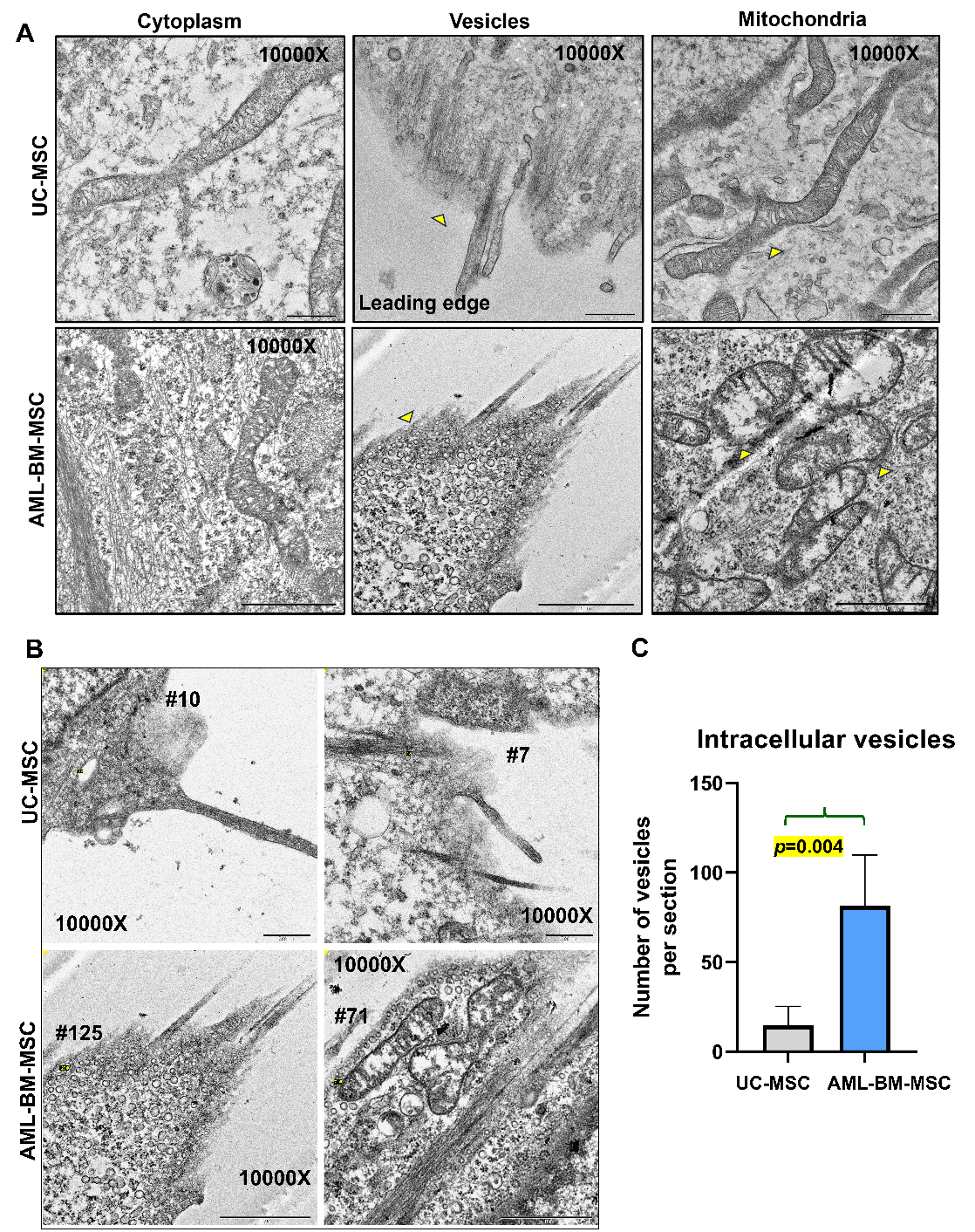


**Fig. S3: Ultrastructural morphological differences in AML-BM-MSC and healthy UC-MSC by TEM.** The electron micrographs of AML-BM-MSC and UC-MSC exhibit variations in terms of **[A]** cytoplasmic density, altered number of cellular vesicles and mitochondrial morphology. **[B]** The values within each panel denote vesicle count. **[C]** Bar graphs exhibit quantification of enumerated intracellular vesicles. The experiment was conducted in 2 biological and 2 technical replicates. All images were captured at a magnification of 10000x. Significant *p*-values are highlighted and accompanied by the Mean ± SEM values. For the enumeration experiment (n=5), different sections were counted for both cell types.

**Supplementary Figure S4**


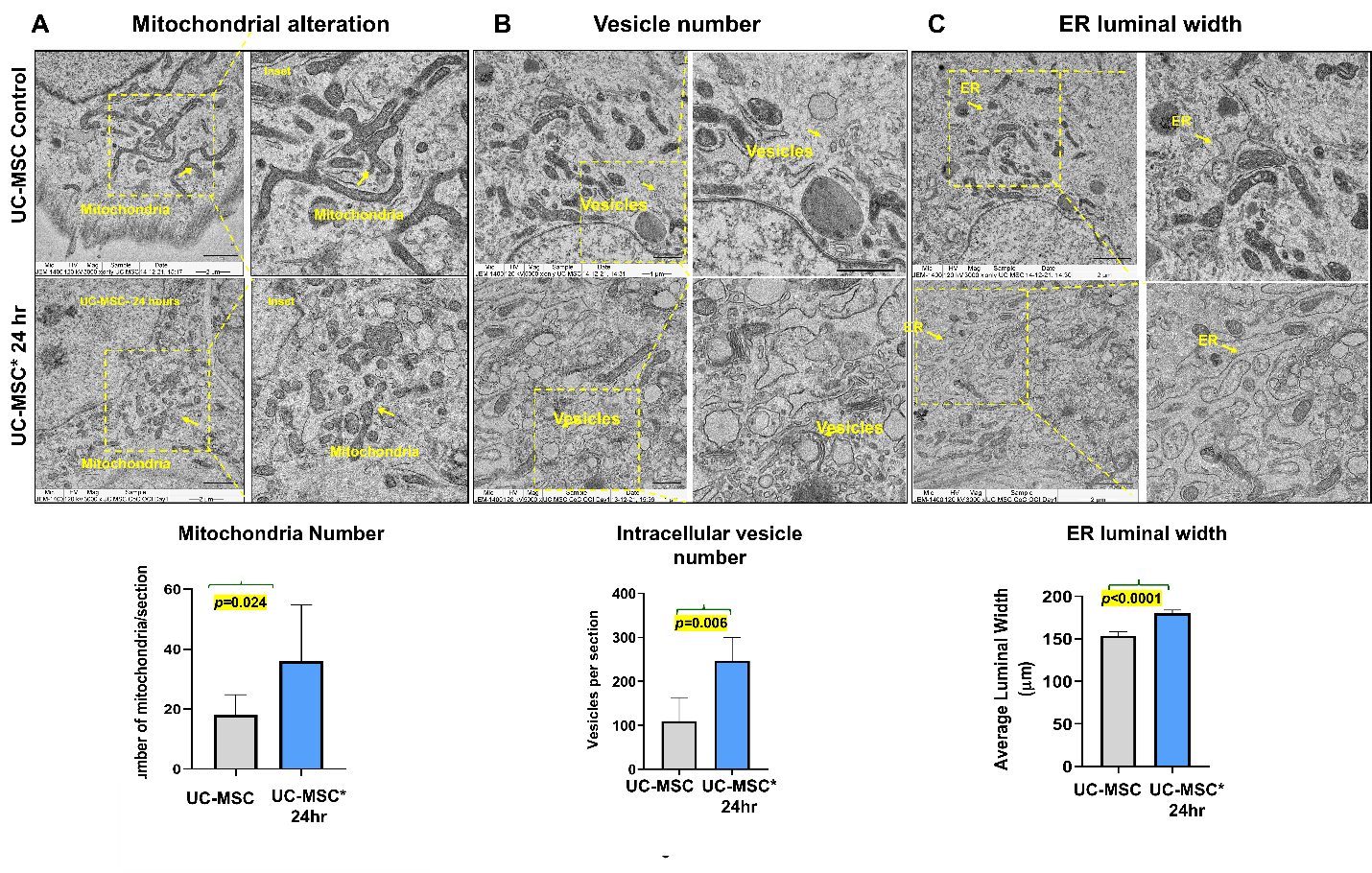


**Fig. S4 Ultrastructural morphological alterations in UC-MSC co-cultured with OCI-AML2 cells.** UC-MSC quantification. **[A]** Mitochondrial number, **[B]** Intracellular vesicle count and **[C]** ER Luminal width in control and co-cultured UC-MSC. Number of fields counted n=3. The experiment was conducted using three biological replicates and two technical replicates. Significant *p*-values are highlighted and accompanied by the Mean ± SEM values. Images were captured and analyzed at 3000x and 5000x, respectively.

**Supplementary Figure S5:**


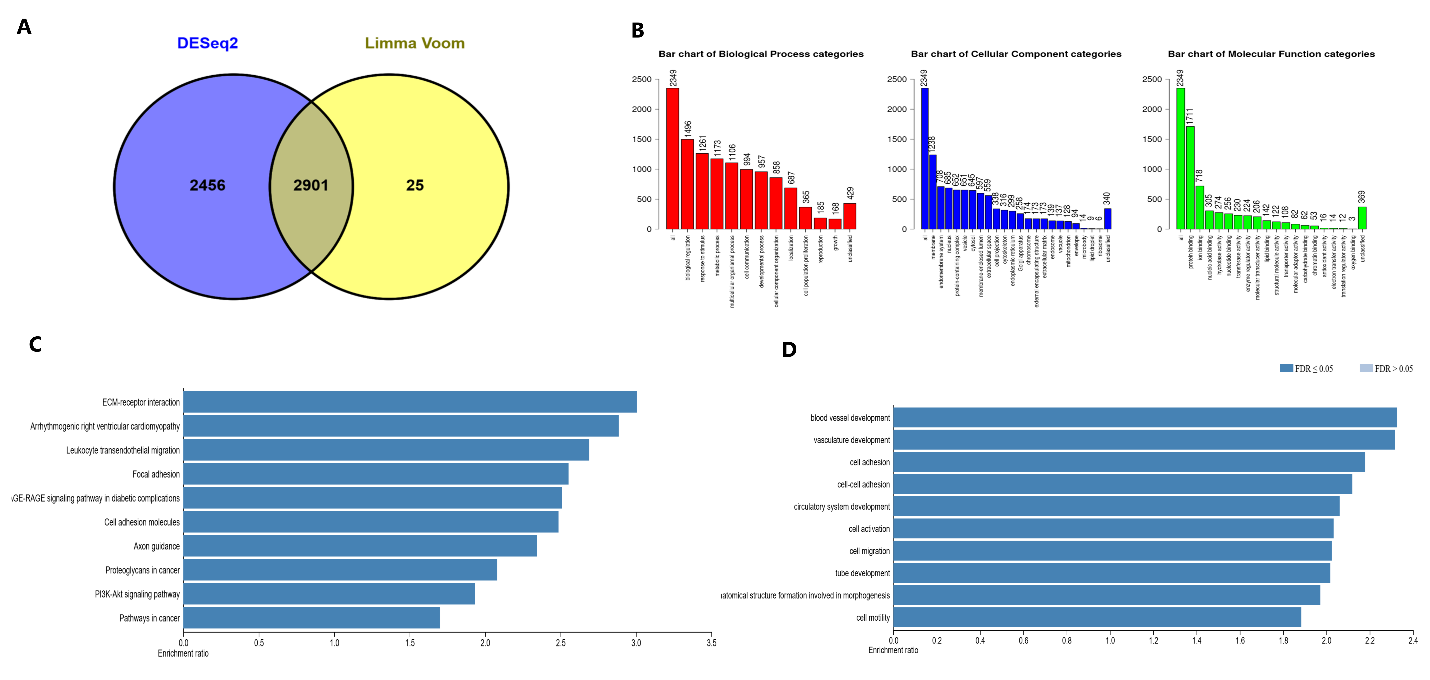


**Fig. S5 Gene expression profiling in AML-BM-MSC** Panel **[A]** shows differential gene expression with Limma-voom package using R software. Overlapping region signifies overlapping genes from both DESeq2 and Limma-voom. Panel **[B]** depicts enriched pathways belonging to biological processes, cellular components and molecular functions. Panel **[C]** shows bar graph of significantly enriched pathway by Over representation analysis (ORA) with FDR $\leq$ 0.05 as significant. Panel **[D]** demonstrates gene ontology conducted to obtain enriched biological processes (BP) with FDR $\leq$ 0.05 as significant.

**Supplementary Figure S6:**

**Fig. S6 Data validation by quantitative real-time PCR.** Bar graph showing fold change difference in inflammasome pathway gene (NLRP3, IL-18, and CASP-1) expression over 18S rRNA in AML-BM-MSC compared to UC-MSC. The experiment was conducted with three technical replicates.

**Supplementary Figure S7**

**
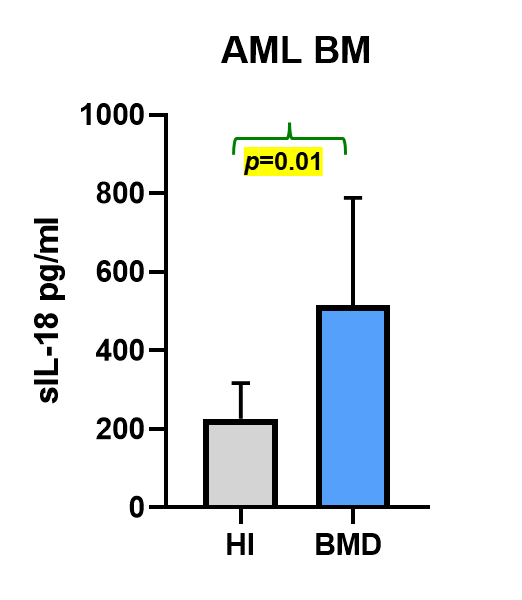
**

**Fig S7: sIL-18 secretion in AML patient bone marrow plasma at diagnosis.** Bar graphs depicting sIL-18 concentration in pg/ml in AML BM plasma (n=4) at diagnosis in comparison to healthy individual peripheral blood plasma (n=5). Significant *p*-values are highlighted in yellow.

**Supplementary Figure 8:**


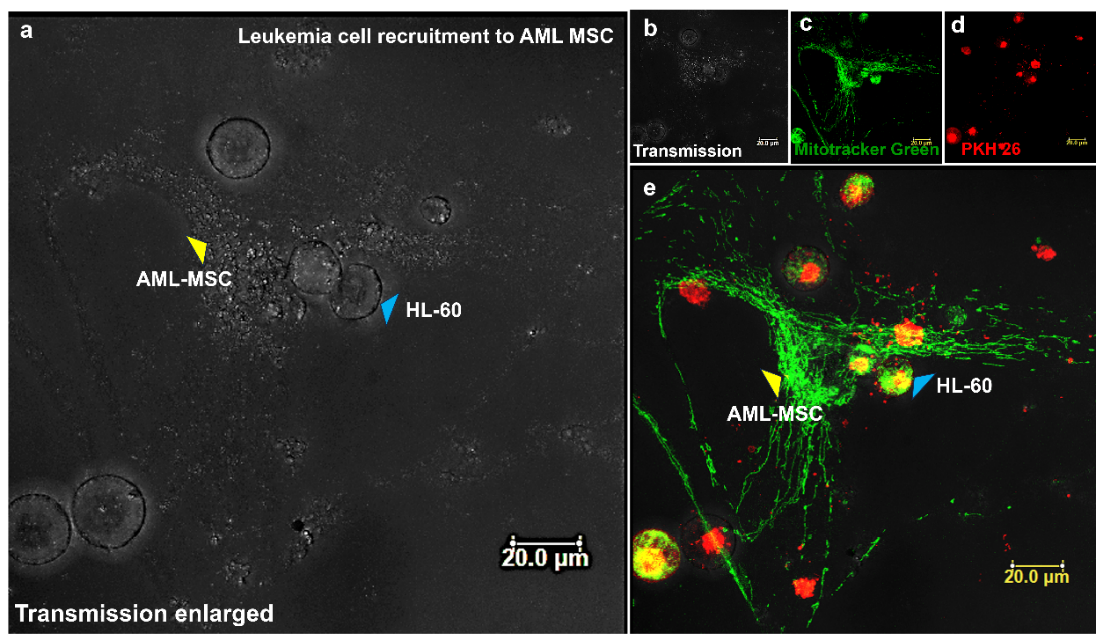


**Fig. S8: Recruitment of HL-60 to AML-MSC** Panel (**a**) demonstrates magnified transmission image showing HL-60 cells (blue arrow) located next to AML-MSC (yellow arrow). Panel (**b**) shows smaller transmission image of that shown in panel (**a**). Panels (**c**) and (**d**) show Mito-tracker green (AML-MSC) and PKH-26 red (HL-60) cells. Leukemic blasts are aligning next to the MSC, ii] Leukemic factors released from HL-60 (Lipid membrane labeled red) being carried through red vesicles are observed with MSC (stained green with mitochondria tracker). Panel (**e**) demonstrates Leukemic blast showing dual color yellow due to transfer of green mitochondria within red labeled HL-60. Images are captured at a scale of 20µm.

**Supplementary Figure S9**


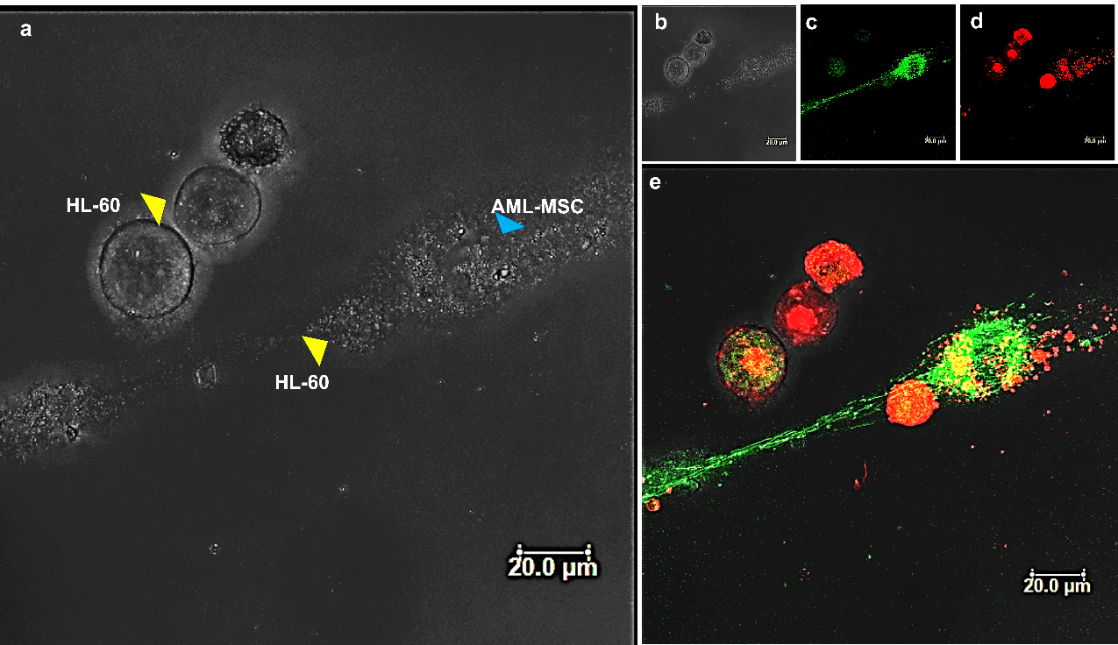


**Fig. S9: Recruitment of HL-60 to AML-MSC** Panel (**a**) demonstrates a magnified transmission image showing HL-60 cells (blue arrow) located next to AML-MSC (yellow arrow). Panel (**b**) shows a smaller transmission image as that of panel a. Panels **(c)** and **(d)** show Mito-tracker green (AML-MSC) and PKH-26 red (HL-60) cells. Leukemic blasts are aligning next to the MSC, ii] Leukemic factors released from HL-60 (Lipid membrane labeled red) being carried through red vesicles are observed with MSC (stained green with mitochondria tracker). Panel **(e)** demonstrates Leukemic blast showing dual color yellow due to transfer of green mitochondria within red labeled HL-60.

**Supplementary Figure S10**


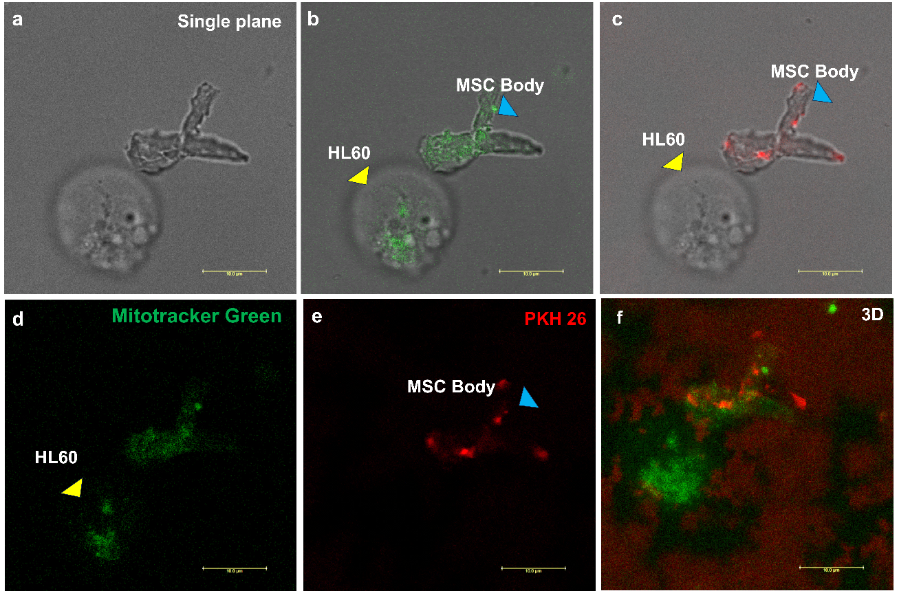


**Fig. S10: Interaction of AML-MSC originating MSC body with leukemia cell (HL-60)** Panel **(a)** demonstrates transmission image of MSC body interacting with HL-60 (leukemia cell line). Panel **(b)** and **(c)** demonstrate merged transmission and stained image of showing HL-60 cells (green) located next to AML-MSC (red). Panel **(d)** and **(e)** show Mito-tracker green stained (HL-60) and PKH26 stained (AML-MSC). Panel **(f)** shows merged image demonstrating MSC body interaction with HL-60 cells. Leukemic blasts are aligning next to the MSC, Leukemic factors released from HL-60 (Lipid membrane labeled red) being carried through red vesicles are observed with MSC (stained green with mitochondria tracker). Panel e demonstrates Leukemic blast showing dual color yellow due to transfer of green mitochondria within red labeled HL-60.

**Supplementary Figure S11:**

**
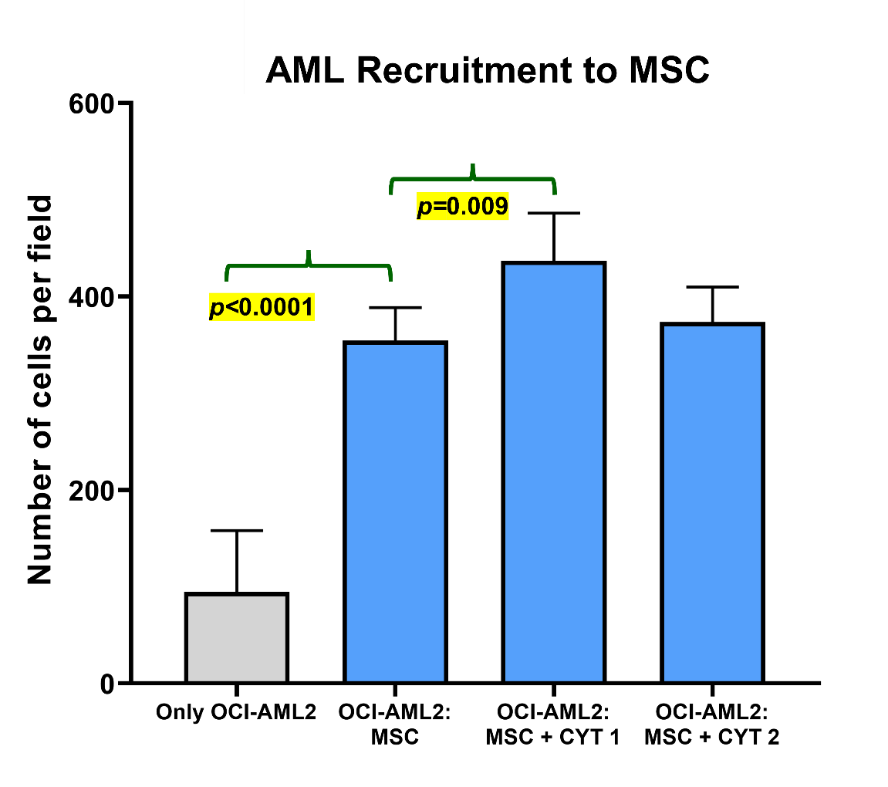
**

**Fig. S11 Trans well migratory potential of OCI-AML2 cells to AML-BM-MSC.** The bar graph illustrates the recruitment of OCI-AML2 to AML-BM-MSC in trans well cultures within 24 hours. *p*-value <0.05 was considered significant and is highlighted in yellow.

**Supplementary Figure S12**


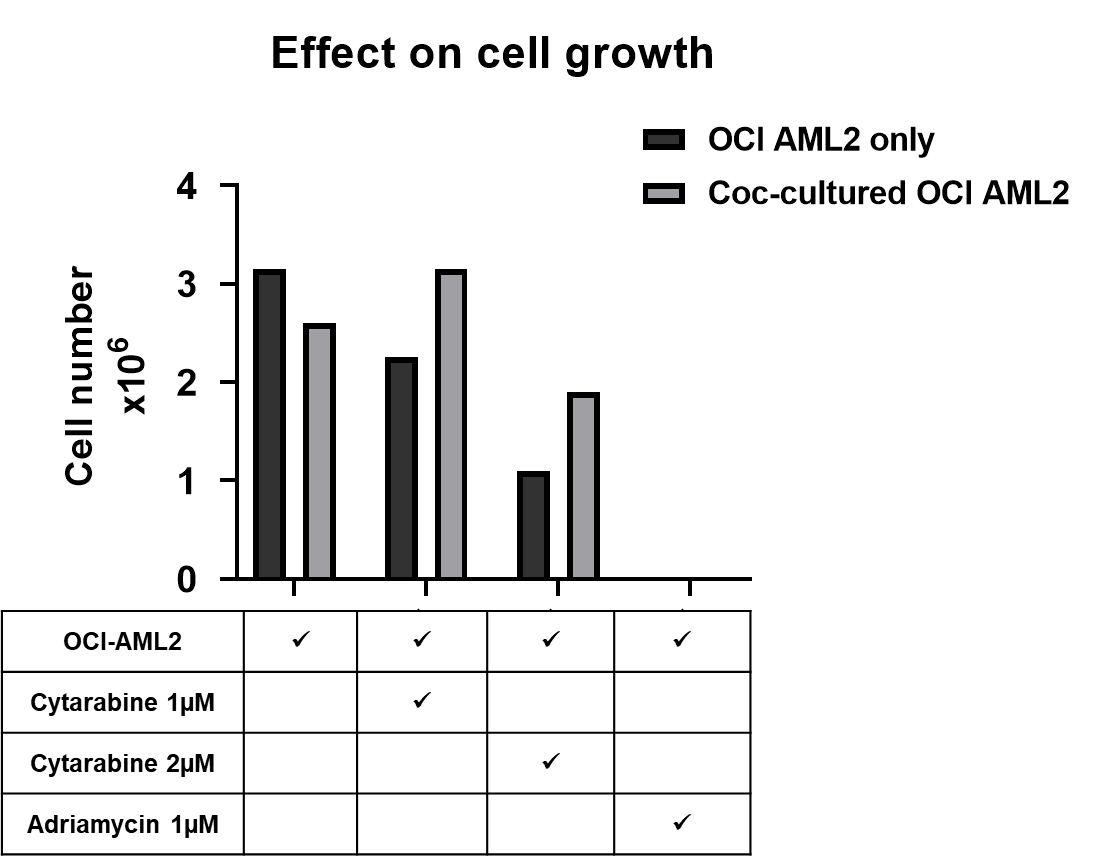


**Fig. S12 Enumeration of viable OCI-AML2 in single and co-cultures** Figure shows representative bar graphs of OCI-AML2 cells cultured in single or in co-cultures with AML-BM-MSC, evaluated for viability by trypan blue staining. The experiment was conducted using two biological replicates.

**Supplementary Figure S13**


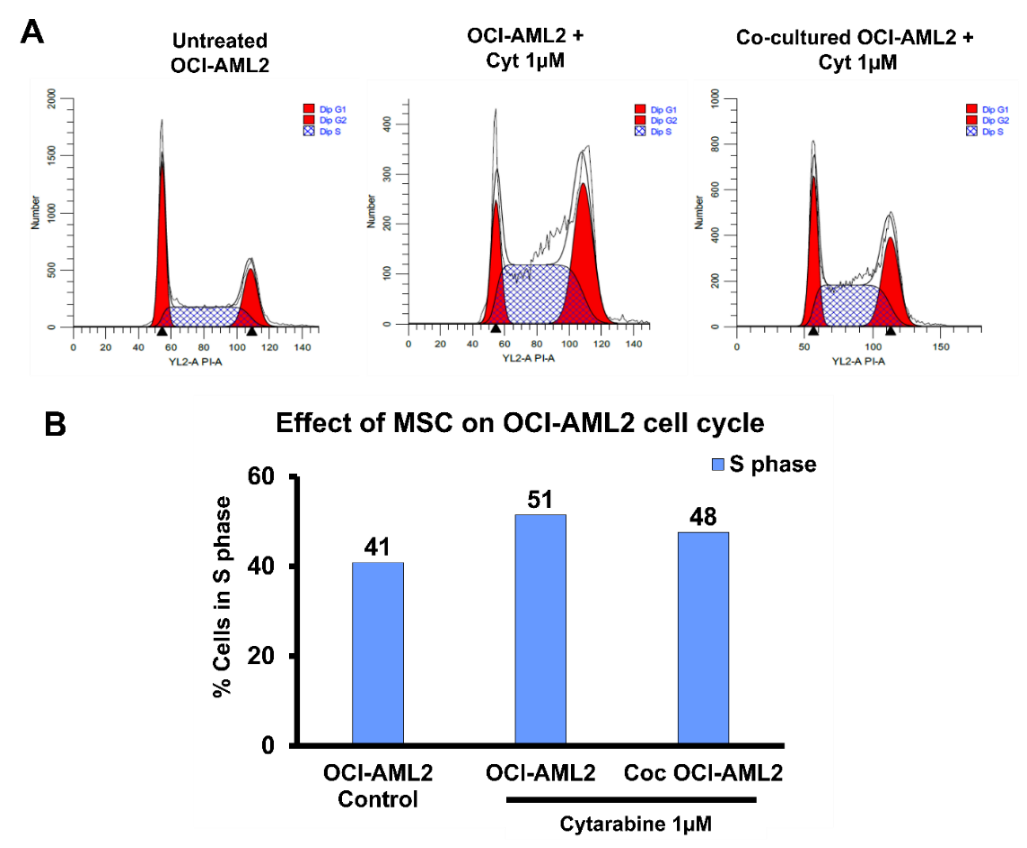


**Fig. S13 Cell cycle modulation effect of OCI-AML2 in response to cytarabine treatment was found to have a marginal effect.**

**Supplementary Figure S14:**

**
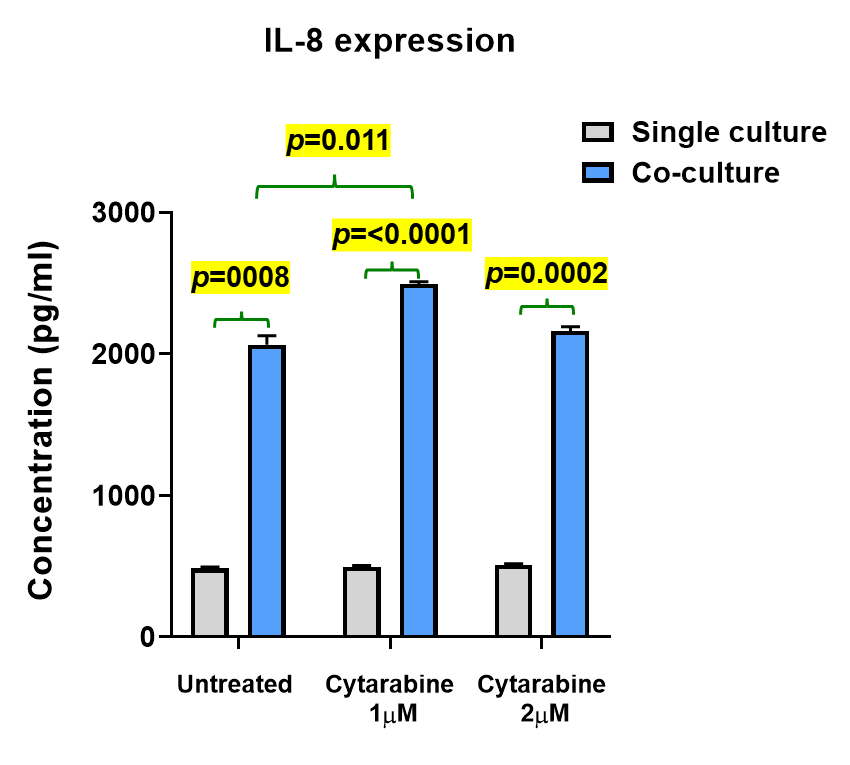
**

**Fig. S14 Soluble IL-8 levels in OCI-AML2 single and co-culture supernatants.** Alteration in cell cycle phase analysis of OCI-AML2 cells as single and co-cultured with AML-BM-MSC for 48 hours in the presence of cytarabine as a standard histogram and bar graph. Significant P-values are highlighted and accompanied by the Mean ± SEM values.

**Supplementary Figure S15:**


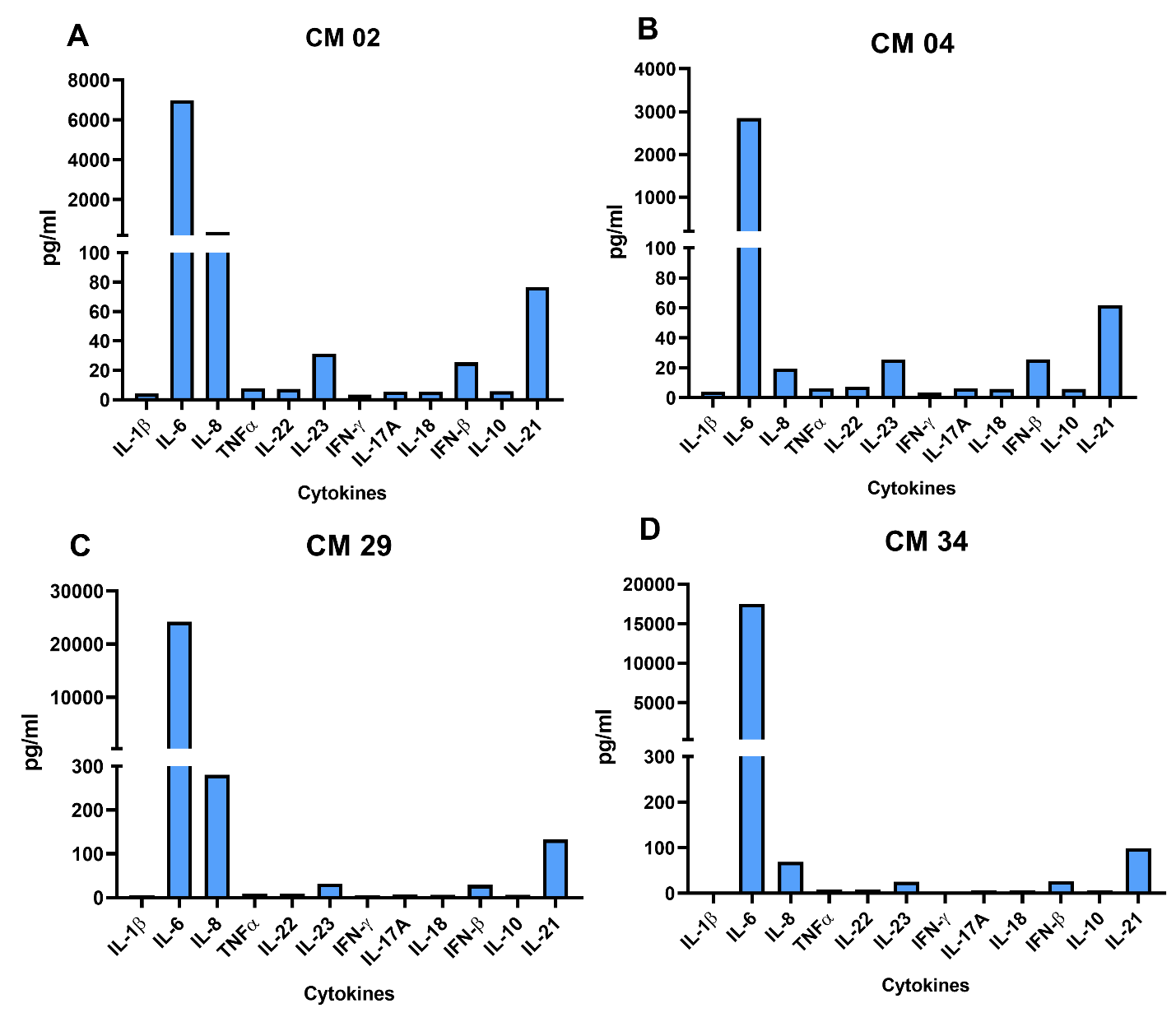


**Fig. S15: PD-MSC-CM composition.** Values are represented in pg/ml

**Supplementary Figure 16:**


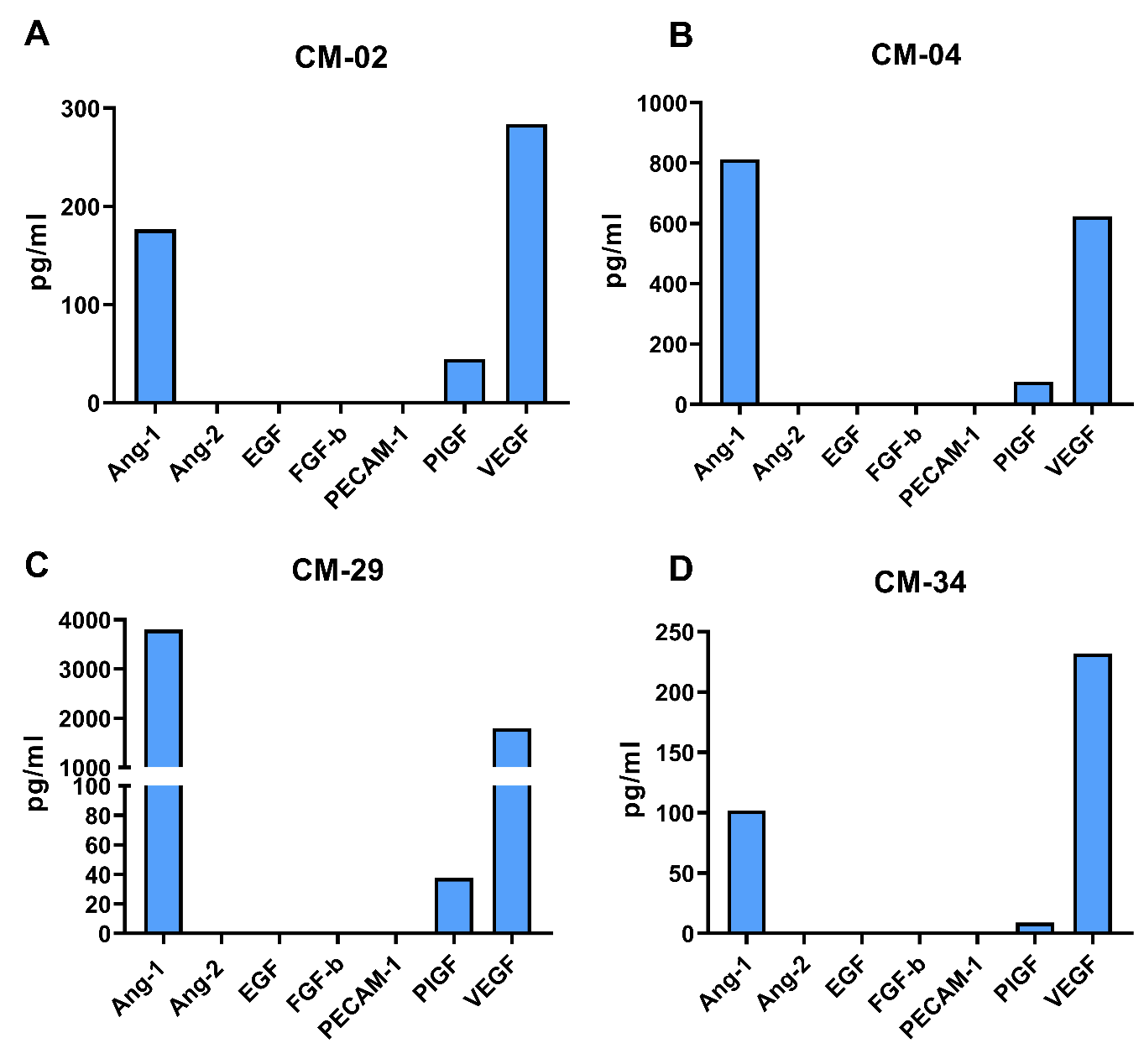


**Fig. S16 PD-MSC-CM composition of growth factors.** Values are represented in pg/ml

**Supplementary Figure 17:**


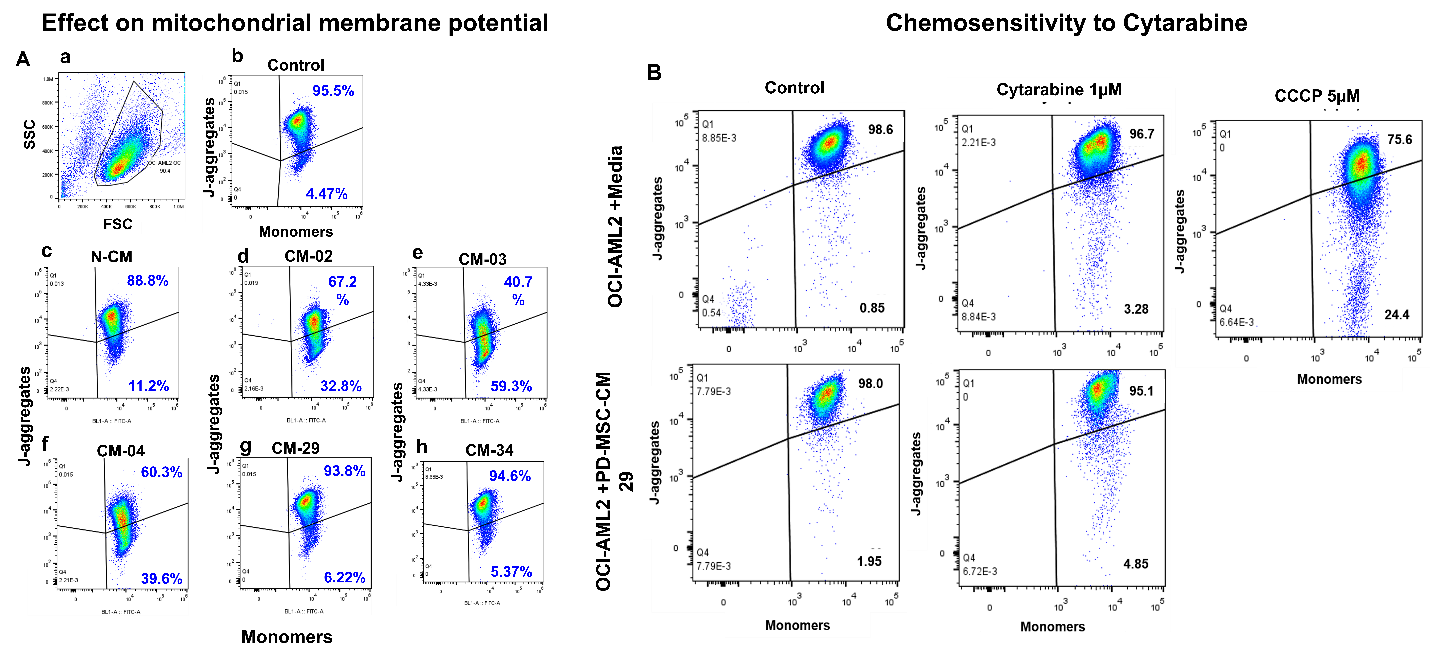


**Fig. S17 Effect of PD-MSC-CM on mitochondrial membrane potential in OCI-AML2 cells [A]** Panel represents the **(a)** FSC vs SSC scatter plots of OCI-AML2 cells. Subpanel **(b)** represents control OCI-AML2 cell scatter gated for dual expression of J-aggregates (y-axis) and Monomers at (x-axis). Subpanels **(c-h)** represent the dual-stained scatter plots of OCI-AML2 cells treated with N-BM-MSC-CM (n=1) and PD-MSC-CM (n=5). **[B]** Mitochondrial membrane potential determination in PD-MSC-CM-34 in the presence of Cytarabine treatment. FSC vs SSC scatter plots of OCI-AML2 cells are depicted as **(a)** control, **(b)** Cytarabine (1µM), **(c)** CCCP (5µM) as a positive control, **(d)** PD-MSC-CM-34 treated and **(e)** PD-MSC-CM-34 in the presence of Cytarabine (1µM).

**Supplementary Figure S18:**


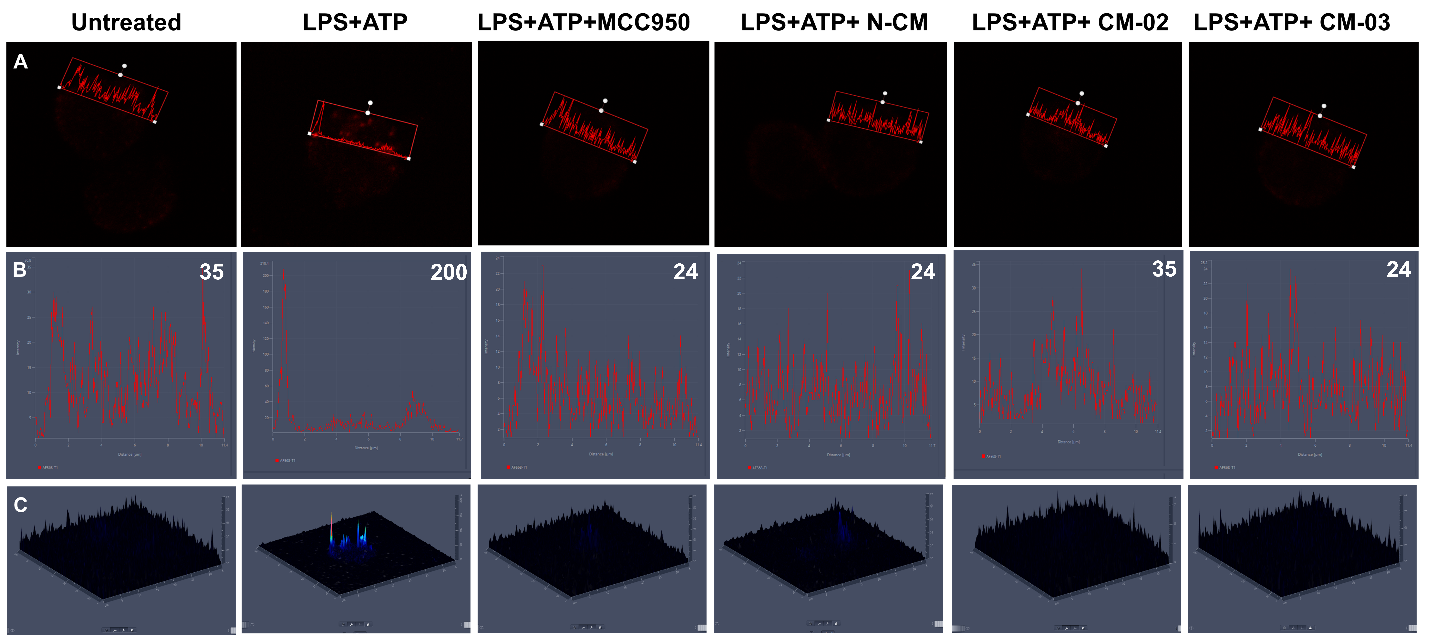


**Fig. S18 OCI-AML2 inflammasome activation model was treated with MCC950 and PD-MSC-CM.** Panel **[A]** depicts cell region with puncta marked for line intensity, **[B]** Line intensity graph where top right values indicated maximum intensity projected, and **[C]** 3D interactive plots of OCI-AML2 cell.

**Supplementary Figure S19:**


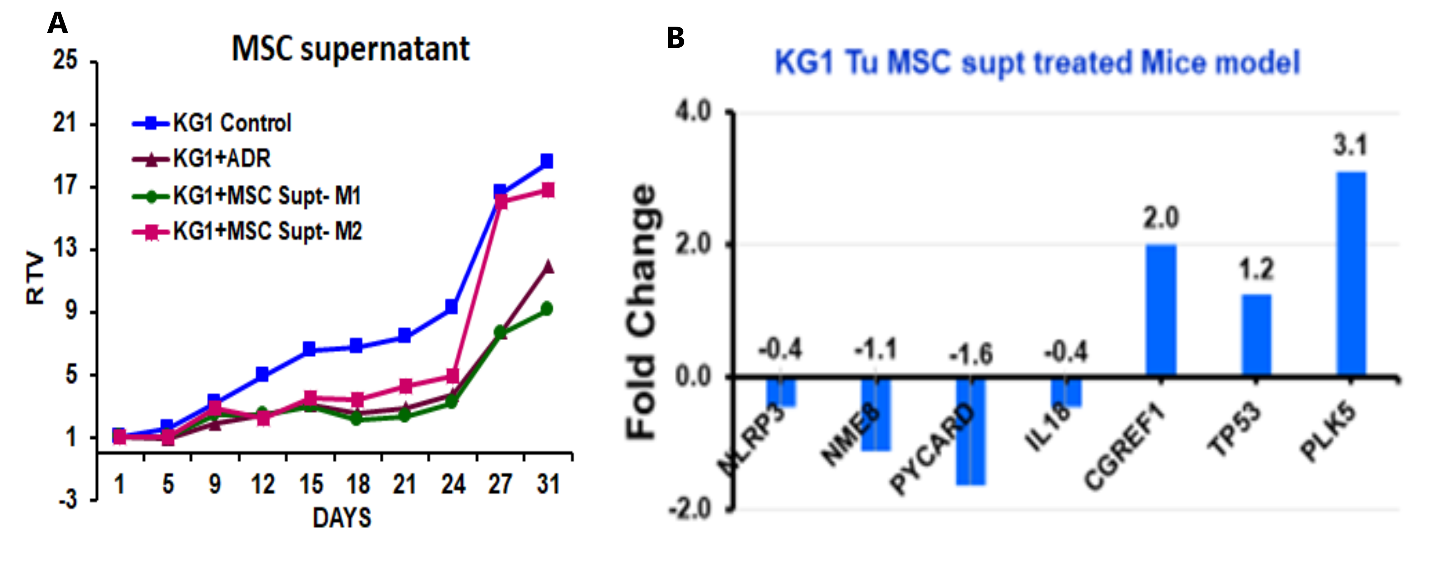


**Fig. S19 In vivo efficacy assay in an immunodeficient mouse model**

Panel **[A]** depicts line graphs for relative tumor volumes of ETP-ALL BM-MSC-CM-treated NOD-SCID mice. The experiment was conducted for 30 days, and tumor volumes were recorded every fourth day. Mice treated with ADR were used as a positive control. Panel **[B]** represents the fold change values of inflammasome pathway gene expressions by microarray analysis.

**Supplementary Video 2**

SV1: Mitochondrial transfer from MSC to AML cells


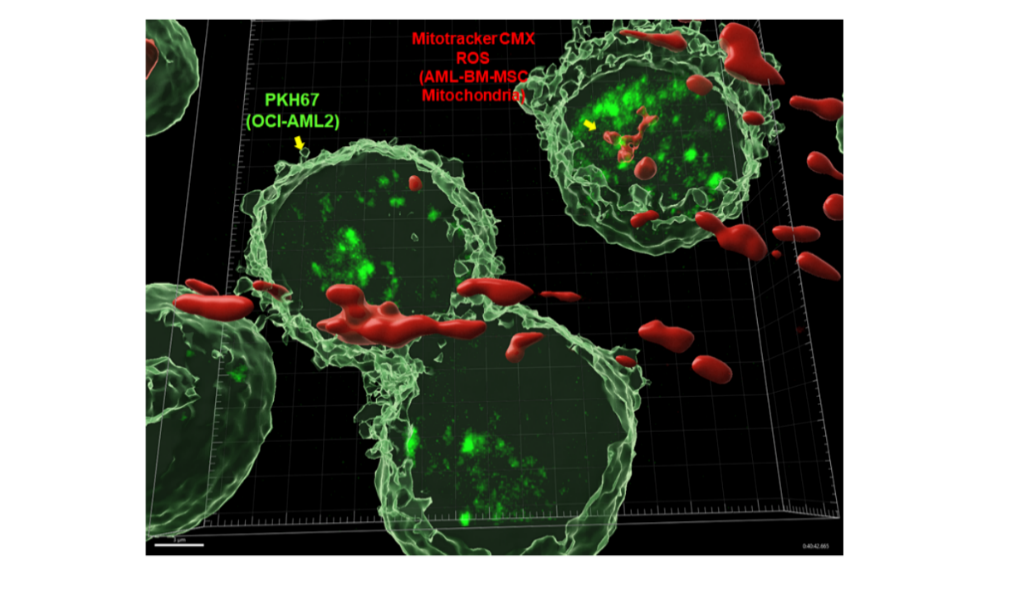


**Supplementary video SV2**

**Evaluation of mitochondrial transfer in AML-BM-MSC to OCI-AML2 cells.** Live mitochondrial transfer between AML-BM-MSC and OCI-AML2 cells is demonstrated in the above figure. Green circles represent the projected images of PKH67-stained OCI-AML2 cells. Red elongated to irregular structures represent the Mito-tracker CMX ROX-stained mitochondria in AML-BM-MSC. The image represents a snapshot of live cell imaging at a scale of 3 µm and 100x magnification by a 3i spinning disc confocal microscope.
