# Supplementary material for: Defining alteration in bone marrow mesenchymal stem cells (MSC) from acute myeloid leukemia and exploring cultured MSC-conditioned media as a novel anti-leukemia therapy agent
Source: Cancer Immunol Immunother. 2026 Feb 23;75(3):83. doi: 10.1007/s00262-025-04262-2 (PMC12929743; doi:10.1007/s00262-025-04262-2)
Supplement: Supplementary file 3 — Supplementary file3 (DOCX 40 KB) [file 262_2025_4262_MOESM3_ESM.docx]

**Defining alteration in bone marrow mesenchymal stem cells (MSC) from acute myeloid leukemia and exploring cultured MSC-conditioned media as a novel anti-leukemia therapy agent**

Manasi Nagare**^1,10^,** Monalisa Sahoo**^1^**, Manju Sengar**^3,10^**, Sachin Punatar**^3,10^**, Navin Khattry**^3,10^**, Anant Gokarn**^3,10^**, Bhausaheb Bagal**^3,10^**, Hasmukh Jain**^3,10^**, Sumeet Mirgh**^3,10^,** Sridhar Epari**^4,10^**, Tanuja Shet**^9,10^**, Trupti Pradhan**^1^**, Shweta Shirsat**^1^**, Madan Barkume**^2^**, Caroline Mathen^11^, Poonam Gera**^7,10^**, Rohit Kumar Verma^6^, Elveera Saldanha^6,10^, Pratik Chandrani^6,10^, Jitendra Gawde^8^, Shubhada Chiplunkar^5,10^, Jyoti Kode**^1,10*^**

**Supplementary Table ST1:** Demographic data for patients included in the study

| Characteristics | Count* | Percent* |
| --- | --- | --- |
|  |  |  |
| **Type** |  |  |
| Healthy | 5 | 100% |
| **Gender** |  |  |
| Male | 5 | 100% |
| Female | 0 | 0% |
| Age Mean (Standard Deviation) | 34.6 ± 10.01 |  |
|  |  |  |
| **Type** |  |  |
| AML | 6 | 67% |
| NHL | 2 | 22% |
| ETP-ALL | 1 | 11% |
|  |  |  |
| **Gender** |  |  |
| Male | 6 | 67% |
| Female | 3 | 33% |
| Age Mean (Standard Deviation) | 54.89 ±14.78 | - |
|  |  |  |
| **CBC counts** |  |  |
| Neutrophils ABS x10^9^/Lit Mean (Standard Deviation) | 5.64 ± 3.91 | - |
| Lymphocytes ABS x10^9^/Lit Mean (Standard Deviation) | 2.52 ± 2.18 | - |
| Monocyte ABS x10^9^/Lit Mean (Standard Deviation) | 0.90 ± 8.45 | - |
| Eosinophils ABS x10^9^/Lit Mean (Standard Deviation) | 0.23 ± 0.23 | - |
| Basophils ABS (Median, IQR) | 0.07 (0.22) | - |
| PLATELETS (Median, IQR) | 61 (99) | - |
| NLR Mean (Standard Deviation) | 2.10 ± 1.55 | - |
| PLR (Median, IQR) | 18.89 (82.21) | - |
| MLR (Median, IQR) | 0.35 (2.34) | - |
|  |  |  |

*Data are expressed in number and percentage, except where stated. # defined as per standard published criteria.

AML- Acute Myeloid Leukemia; NHL- Non-Hodgkin Lymphoma; ETP-ALL- Early T-cell precursor Acute Lymphoid Leukemia; NLR-Neutrophil Lymphocyte Ratio; MLR- Monocyte Lymphocyte Ratio; PLR- Platelet Lymphocyte ratio; ABS- Absolute; IQR- Interquartile Range;

**Supplementary Table ST2: List of reagents and**

| **Sr No.** | **Reagent name** | **Company** | **Catalogue no.** | **Conjugate** |
| --- | --- | --- | --- | --- |
| **1.** | **Blood sample processing** |  |  |  |
| a | Ficoll | Sigma | F4375 |  |
| b | Sodium diatrizoate | Sigma | S4506 |  |
|  |  |  |  |  |
| **2.** | **Rosette Sep Human Mesenchymal stem cell enrichment cocktail** | Stem cell technologies, Canada | 15128 |  |
|  |  |  |  |  |
| **3.** | **Human mesenchymal stem cell stimulatory supplement** | Stem cell technologies | 05402 |  |
|  |  |  |  |  |
| **4.** | **Immunophenotyping** |  |  |  |
| i. | CD 45 | Milteny Biotech |  | FITC |
| ii. | CD 90 | Bio Legend | 555597 | PE CY5 |
| iii. | CD 73 | Bio Legend | 344101 | PE CY7 |
| iv. | CD 105 | Bio Legend | 560819 | PERCP CY5.5 |
| v. | TIM-3 | Bio Legend | 565565 | BV650 |
| vi. | TLR3 | Bio Legend | 315010 | PE |
| vii. | TLR4 | Bio Legend | 312811 | BV421 |
| viii. | A2AR | Bio Legend |  | APC |
| ix. | A2BR | Bio Legend |  | AF488 |
|  |  |  |  |  |
| 3. | **Reagents** |  |  |  |
| i. | Paraformaldehyde | Sigma | P6148 |  |
| ii. | Sodium Azide | Sigma | 26628-22-8 |  |
| iii. | Trypan blue | Sigma | T6146 |  |
|  |  |  |  |  |
| **4** | **Cytokine bead array** | BD Biosciences | 551811 |  |
|  |  |  |  |  |
| **5** | **AimPlex^TM^ Premixed Multiplex Kit** | Biosciences.Inc | T1C1240355 |  |
|  |  |  |  |  |
| 6 | **LEGEND Plex** | BioLegend | 741214 |  |
|  |  |  |  |  |
| **7** | **Fluorescent staining dyes** |  |  |  |
| a | Mito Tracker CMX ROS | Invitrogen | M46752 |  |
| b | Mito Tracker FM green | Invitrogen | 2411518 |  |
| c | JC-1 | Invitrogen | CBIC2(3) |  |
| d | PKH67 green | Sigma | MIDI67 |  |
| e | PKH26 red | Sigma | MIDI26 |  |
|  |  |  |  |  |
| **8** | **Medium** |  |  |  |
| a | MEM-alpha | Gibco, India | 11900-24 |  |
| b | DMEM | Gibco, India | 12800-017 |  |
| c | IMDM | Gibco, India | 12200-036 |  |
| d | HEPES | Sigma | H/3784 |  |
|  |  |  |  |  |
| **9** | **Cell growth estimation** |  |  |  |
| a | MTT | Sigma | M-2128 |  |
| b | SRB | Sigma | S9012 |  |
|  |  |  |  |  |
| **10** | **Immunohistochemistry** |  |  |  |
| a | Envision FLEX Mini Kit | Dako | K-8023 |  |
| b | IL-1β | Invitrogen | Abcam |  |
| c | NLRP3 | Invitrogen | MA5-32255 |  |
| d | Caspase-1 | Invitrogen | MA5-16215 |  |
|  |  |  |  |  |
| **11** | **Lineage differentiation** |  |  |  |
| a | Mesencult Basal Medium | STEMCELL Technologies | 05402 |  |
| b | Adipogenic stimulatory supplement | STEMCELL Technologies | 05412 |  |
| c | Osteogenic stimulatory supplement | STEMCELL Technologies | 05465 |  |
|  |  |  |  |  |
| **12** | **Cell cycle** |  |  |  |
| a | RNase A | Sigma | R6513 |  |
| b | Propidium Iodide | Sigma | 4170 |  |
|  |  |  |  |  |
| **13** | **RNA extraction** |  |  |  |
| a | TRIzol | Invitrogen | 15596026 |  |
| b | Chloroform | Merck | 1024451000 |  |
| c | Isopropanol | Merck | [W292907](https://www.sigmaaldrich.com/IN/en/product/aldrich/w292907) |  |
| d | DEPC | Sigma | D5758 |  |
|  |  |  |  |  |
| **14** | **DNase I kit** | Molecular biology | EN0521 |  |
|  |  |  |  |  |
| **15** | **cDNA synthesis** | Molecular biology | K1632 |  |
|  |  |  |  |  |
| 16 | **Sybr Green q PCR kit** | Kappa Biosystems |  |  |
|  |  |  |  |  |

**Supplementary Table ST3: Real-time PCR primer list**

| Primer | Forward Primer (5’-3’) | Reverse Primer (5’-3’) |
| --- | --- | --- |
| NLRP3 | 5’- ATG AGC CGA AGT GGG GTT -3’ | 5’- GTG TGT AGC GTT TGT TGA GG -3’ |
| IL-18 | 5 AGA GGT ATG GCT GTA ACT AT 3’ | 5 ATG TCA CTT TTT GTA TCC TTG 3’ |
| Caspase-1 | 5’- ATC TCA CTG CTT CGG ACA -3’ | 5’- GTA TAT CTG GGA CTT GCT CA -3’ |
| 18S rRNA | 5'- GAT GGT AGT CGC CGT GCC- 3' | 5'- GCC TGC TGC CTT CCT TGG- 3' |

**Supplementary Table ST4:**

| **Cytokines**  **(pg/ml)** | **N-BM-MSC-CM** | **PD-MSC-CM-02** | **PD-MSC-CM-03** | **PD-MSC-CM-04** | **PD-MSC-CM-29** | **PD-MSC-CM-34** |
| --- | --- | --- | --- | --- | --- | --- |
| IL-1β | 4.69 | 4.23 | 4.41 | 4.16 | 4.53 | 4.1 |
| IL-6 | 2086.98 | 6976.02 | 5908.38 | 2850.44 | 24173.56 | 17505.82 |
| IL-8 | 8.37 | 395.93 | 477.73 | 19.6 | 280.03 | 68.57 |
| TNF-α | 7.23 | 7.61 | 8.52 | 6.48 | 8.39 | 7.69 |
| IL-22 | 8 | 7.48 | 7.23 | 7.53 | 8.1 | 7.59 |
| IL-23 | 28.62 | 31.37 | 24.13 | 25.46 | 31.74 | 24.83 |
| IFN-γ | 3.43 | 3.57 | 2.85 | 3.77 | 4.55 | 3.43 |
| IL-17A | 6.59 | 5.56 | 5.98 | 6.31 | 6.78 | 6.11 |
| IL-18 | 5.37 | 5.33 | 5.39 | 5.94 | 6.65 | 5.57 |
| IFN-β | 27.29 | 25.4 | 26.22 | 25.73 | 29.01 | 25.92 |
| IL-10 | 6.37 | 5.77 | 6.05 | 6.01 | 6.38 | 5.77 |
| IL-21 | 46.08 | 76.58 | 81.43 | 61.8 | 132.79 | 98.4 |

IL-1β- Interleukin 1-beta; IL-6- Interleukin-6; IL-8- Interleukin 8; TNF-α – Tumor Necrosis Factor- alpha; IL-22; IL-23; IFN-γ; EGF- Epithelial Growth Factor; FGF- Fibroblast Growth Factor; CXCL8- C-X-C motif chemokine ligand 8, PECAM-1 - Platelet Endothelial Cell Adhesion Molecule-1, PlGF – Placental Growth Factor; VEGF- Vascular Endothelial Growth Factor; BDL-below detection levels.

**Supplementary Table ST5**

| **Growth Factors (pg/ml)** | **N-BM-MSC-CM** | **PD-MSC-CM-02** | **PD-MSC-CM-03** | **PD-MSC-CM-04** | **PD-MSC-CM-29** | **PD-MSC-CM-34** |  |
| --- | --- | --- | --- | --- | --- | --- | --- |
|  |  |  |  |  |  |  |  |
| IL-6 | 335.05 | 1430.29 | 729.065 | 1421.895 | 4696.39 | 1152.735 |  |
| Angiopoietin-1 | 141.9 | 176.7 | 311.5 | 812.75 | 3802.8 | 101.7 |  |
| Angiopoietin-2 | BDL | BDL | BDL | BDL | BDL | BDL |  |
| EGF | BDL | BDL | BDL | BDL | BDL | BDL |  |
| FGF- basic | BDL | BDL | BDL | BDL | BDL | BDL |  |
| CXCL8 | 7.8 | 82.08 | 104.165 | 44.51 | 96.075 | 90.425 |  |
| PECAM-1 | BDL | BDL | BDL | BDL | 39.75 | BDL |  |
| PlGF | 7.65 | 44.7 | 44.6 | 74.8 | 37.6 | 8.85 |  |
| VEGF | 90.4615 | 283.899 | 271.854 | 624.355 | 1793.553 | 232.15 |  |
| TNF-α | BDL | 5.126 | BDL | BDL | BDL | BDL |  |

IL-6- Interleukin-6; EGF- Epithelial Growth Factor; FGF- Fibroblast Growth Factor; CXCL8- C-X-C motif chemokine ligand 8, PECAM-1 - Platelet Endothelial Cell Adhesion Molecule-1, PlGF – Placental Growth Factor; VEGF- Vascular Endothelial Growth Factor; TNF-α – Tumor Necrosis Factor- alpha; BDL-below detection levels.
